# Robust Radiative Cooling in Functionalizable Silica Microsphere Paints

**DOI:** 10.1021/acsphotonics.6c01355

**Published:** 2026-10-19

**Authors:** Jorge Burgos, José Rodrigo Magana, Ares Lladós, Javier Pascualena Ferré, Sara Núñez-Sánchez, Juliana Jaramillo Fernández, Cefe López, Pedro David García

**Affiliations:** † 69570 Instituto de Ciencia de Materiales de Madrid (ICMM), Consejo Superior de Investigaciones Científicas (CSIC), Calle Sor Juana Inés de la Cruz 3, 28049 Madrid, Spain; ‡ Cooling Photonics Sociedad Limitada, 08022 Barcelona, Spain; § Departament de Màquines i Motors Tèrmics, Universitat Politècnica de Catalunya, 08028 Barcelona, Spain

**Keywords:** radiative cooling, passive daytime cooling, photonic glass, multiple light scattering, transport mean free path, silica microspheres

## Abstract

Disordered coatings based on silica microspheres provide a scalable and robust platform for passive daytime radiative cooling. While particle-size optimization is often considered critical for enhancing solar scattering, the role of microsphere diameter once a coating operates in the multiple-scattering regime remains unclear. Here, we characterize the radiative cooling performance of disordered, multiply scattering photonic glass coatings with diameters ranging from 2 to 8 μm, deposited on a highly reflective substrate. Despite measurable differences in microscopic scattering properties, both the spectral radiative response and the net cooling performance are robust to variations in particle diameter within the investigated range. Outdoor thermal measurements reveal nearly identical steady-state temperature reductions across the full size range. These results identify this robustness as a property of the complete coating–substrate architecture: in the solar range, multiple scattering and the reflective substrate act together to maintain high reflectance, while in the mid-infrared range, the response is dominated by the intrinsic Si–O absorption of the silica layer. This robustness enables microsphere size to be selected based on surface chemistry or processing constraints without compromising cooling performance.

## Graphical abstract

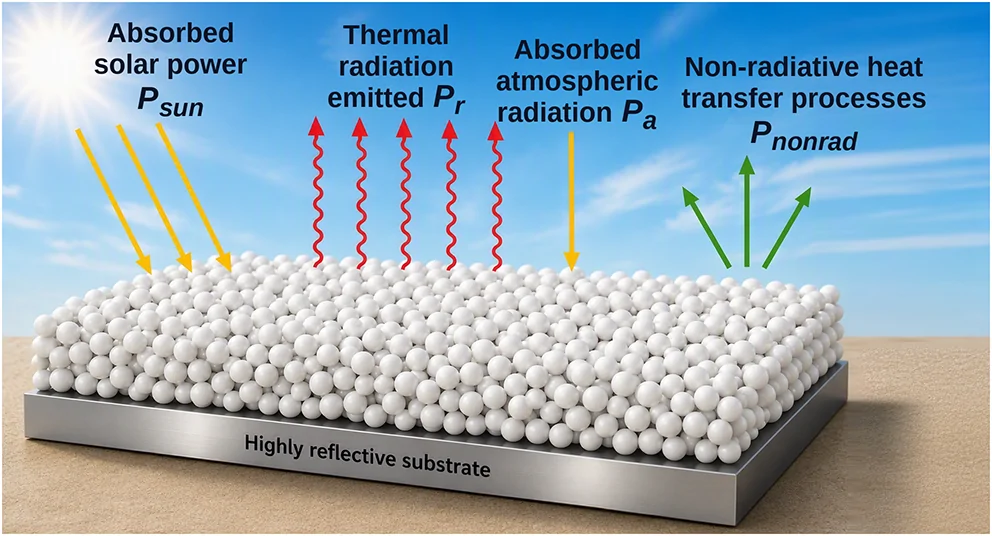

## Introduction

Radiative cooling (RC) enables surfaces to dissipate heat into outer space through the atmospheric transparency window (ATW, 8–13 μm), a spectral region where atmospheric absorption is minimal. For effective below-ambient daytime cooling, materials must combine solar reflectance above 90% (0.3–2.5 μm) with strong thermal emission in the ATW range, such that radiative losses to the cold sky outweigh conductive, solar, and atmospheric heat gains.^1–4^ Silica (SiO_2_) microspheres meet both requirements: their wide bandgap minimizes solar absorption, while Si–O vibrational modes provide strong mid-infrared emission in the ATW.^5–7^ In disordered packings of these spheres, multiple Mie scattering enhances solar reflectance while the resulting porous structure boosts thermal emissivity,^8^ enabling the performance levels summarized in Table 1. Light in such coatings undergoes many scattering events before exiting, a regime characterized by a transport mean free path $l_{t}$ .^7,9^ When coatings are much thicker than $l_{t}$ , transport becomes diffusive and collective, even resonant if their diameter polydispersity is low^10^—yet whether microsphere diameter matters for radiative cooling in such multiply scattering coatings remains unexplored. Prior work optimized particle size for single-scattering efficiency, but it is not obvious that such optimization remains relevant once collective multiple scattering dominates.

**Table 1:** Summary of Radiative Cooling Performance for Different SiO_2_-Based Material Configurations Reported in the Literature

Configuration	Particle size	Thickness	Temperature drop	Solar reflectance^d^	Emissivity	Cooling power	ref
	(μm)	(μm)	(°C)	(%)		(W m^–2^)	
Randomly distributed in TPX polymer matrix^a^	∼4	50	–	∼96	>0.93	93	Zhai et al.^6^
Randomly packed	2	700	12	n.r.^e^	>0.94	–	Atiganyanun et al.^7^
Raspberry-like hollow spheres embedded in PDMS^b^	1	2000	9.7	n.r.^e^	>0.85	114	Park et al.^11^
Ordered self-assembled monolayer^c^	8	8	19	–^f^	0.98	160–350	Jaramillo et al.^12^
Densely packed TiO_2_ (top) and SiO_2_ (bottom)	<0.3	–	8	90.7	0.90	–	Bao et al.^13^
SiO_2_ randomly distributed in PMMA matrix	8	1400	12	93	∼0.89	–	Tian et al.^14^
Porous cellulose-acetate/SiO_2_ film	n.r.^e^	150 (SiO_2_-rich layer 40)	6.2 (day), 8.6 (night)	96	0.95	–	Xiang et al.^15^
SiO_2_ microsphere monolayer on SiO_*x*_N_*y*_/Ag stack	2	monolayer on 1.5–4 SiO_*x*_N_*y*_	5 (autumn), 2.5 (summer)	96.4	0.946	16^g^	Lin et al.^16^
Transparent aggregated SiO_2_/PDMS film on PV module	3 (aggregates of 0.12 μm spheres)	600	7.5 (spring), 6.9 (summer)^h^	–^f^	0.91–0.92	61^i^	Le et al.^17^

a Average cooling power measured around noon at Δ*T* = 0; subambient temperature reductions were also reported.

b Theoretically calculated net cooling power at Δ*T* = 0.

c Measured onto silicon substrates and at Δ*T* from 25 up to 50 °C, showing a cooling power of 70 W m^–2^ at Δ*T* = 0.

d Solar reflectance values are integrated over the wavelength ranges reported in the respective references, which differ among studies.

e Not reported: Atiganyanun et al. state that reflectometry on their strongly scattering coatings was not sufficiently accurate and report a calculated solar absorptivity instead; Park et al. report spectral reflectivity over 0.4–2.5 μm without a solar-weighted value; Xiang et al. use Stöber microspheres without a stated diameter.

f Deliberately translucent or transparent designs intended for cooling of solar absorbers or photovoltaic panels; an integrated solar reflectance is not a reported figure of merit.

g Calculated noontime cooling power under MODTRAN tropical atmospheric conditions.

h Maximum reduction relative to a bare photovoltaic module and not to ambient air.

i Calculated net cooling power at a film temperature of 328 K.

Here, we address this question by characterizing photonic glass coatings with diameters ranging from 2 to 8 μm. This range was selected because it covers the regime in which micrometer-scale silica particles become efficient thermal emitters in the atmospheric window: across 8–13 μm, diameters of 2–8 μm span size parameters from approximately 0.5 to 3, covering the resonant regime in which thin or ordered silica structures exhibit a pronounced diameter dependence of thermal emission,^12,18^ and it also matches the particle sizes commonly employed in the SiO_2_-based radiative coolers of Table 1. Combining optical spectroscopy, coherent backscattering measurements, and outdoor thermal characterization, we show that both spectral response and net cooling performance are largely insensitive to variations in particle diameter for the complete coating–substrate architecture studied here. These results identify this robustness as an empirical property of reflective-substrate-backed, multiply scattering coatings over the investigated range, enabling microsphere size to be selected based on surface chemistry or processing constraints—particularly relevant since the micrometer range offers versatile colloidal synthesis and functionalization routes^19^ and lifts strong constraints relating to colloidal assembly. Figure 1 illustrates the morphology of the fabricated coatings, prepared following the photonic glass approach introduced in ref^10^; the disordered packing of the microspheres is represented schematically in Figure 1(a). Coatings with nominal microsphere diameters of 2, 4, 6, and 8 μm were deposited onto substrates with solar reflectance of 96.3% over 250–2500 nm [Figure 2(a)]. Scanning electron microscopy [Figures 1(b,c) and S1] reveals uniform coatings with randomly arranged particles; the approximately 25 μm thickness was determined by mechanical profilometry, while optical images [Figure 1(d)] confirm homogeneous macroscopic coverage of the complete 5 × 5 cm^2^ substrates without visible defects or particle aggregation.

**Figure 1:**
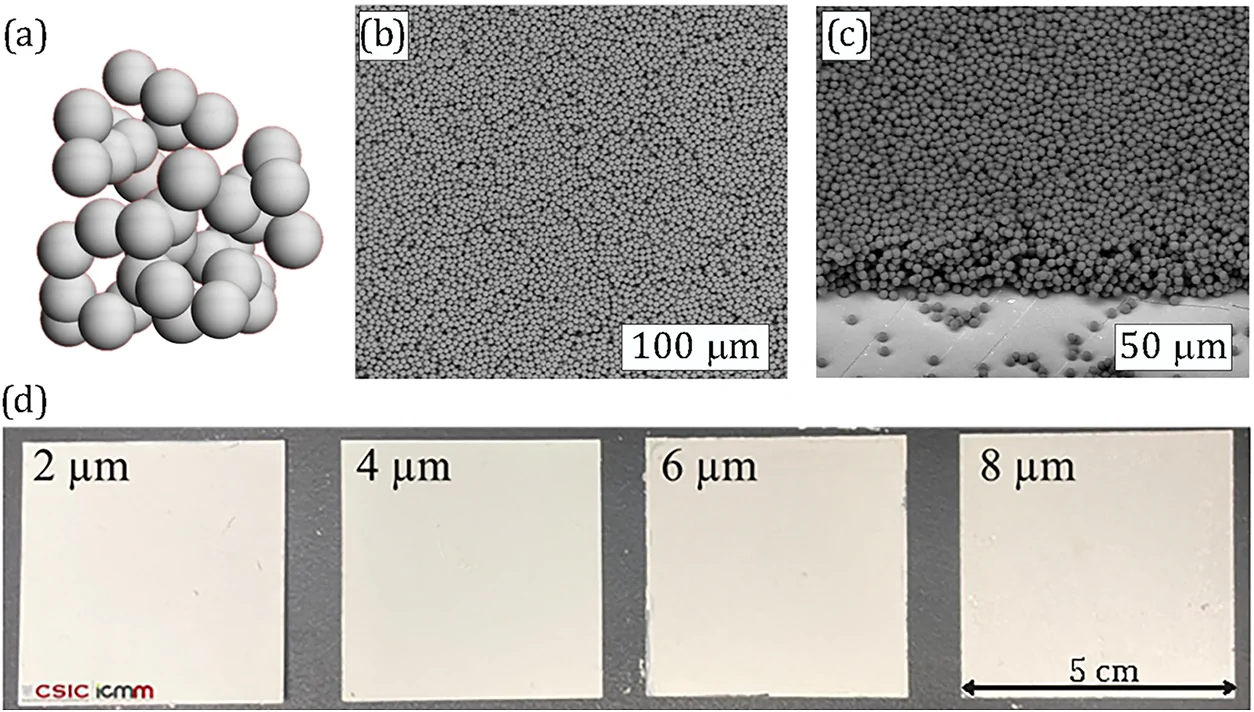
Morphology and structure of photonic glass coatings. (a) Schematic representation of randomly packed silica microspheres forming the disordered photonic medium. (b) Top-view SEM image of the 4 μm coating showing uniform particle distribution. (c) Cross-sectional SEM image showing the coating morphology across its thickness. (d) Optical images of all four coatings (2, 4, 6, 8 μm) showing homogeneous macroscopic coverage of the 5 × 5 cm coatings.

**Figure 2:**
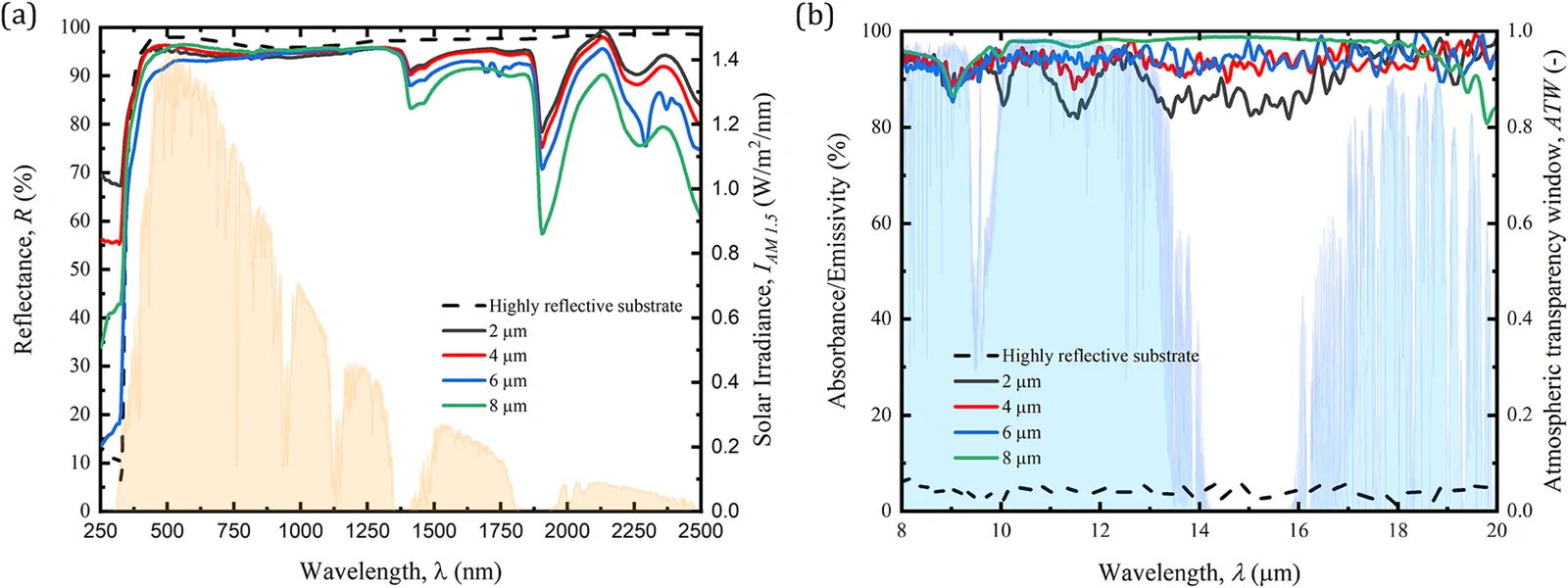
Optical characterization of photonic glass coatings. (a) Reflectance spectra measured over the full 250–2500 nm solar range for all coatings and the bare substrate, overlaid with the AM1.5 global solar irradiance spectrum. The AM1.5-weighted solar reflectance is 93.8–94.8% for the coatings and 96.3% for the bare substrate, sufficient to minimize solar absorption. (b) Mid-infrared emissivity spectra (8–20 μm) with the atmospheric transparency window highlighted. The silica coatings achieve an emissivity of 0.91–0.95 in the 8–13 μm atmospheric window, in contrast to the bare substrate (*ε* ≈ 0.02), enabling efficient thermal radiation to outer space.

## Results and Discussion

The spectral radiative properties relevant to cooling performance of the photonic glass coatings are shown in Figure 2. Solar reflectance spectra measured over the full 250–2500 nm solar range [Figure 2(a)] show the high reflectance of all coatings. The solar reflectance, obtained by weighting the measured spectra with the AM1.5 global solar irradiance over 250–2500 nm, is 94.1%, 94.5%, 94.8%, and 93.8% for the 2, 4, 6, and 8 μm coatings, respectively, and 96.3% for the bare substrate, corresponding to a solar absorptance of 5.2–6.2% for the coatings. The spectra reveal the expected reflectance reduction in the near-infrared, associated with water- and hydroxyl-related absorption features of the silica coatings near 1.4 and 1.9 μm and beyond 2.2 μm; because the solar irradiance is comparatively low at these wavelengths, their weight in the AM1.5 integral is limited. Restricting the integration to 250–900 nm yields 93.9–95.0% for the four coatings. The particle diameter affects the spectra mainly in the near-infrared. Between 400 and 1300 nm, where most of the solar irradiance lies, the four coatings are indistinguishable within approximately 2% and show no ordering by diameter. The reflectance minima near 1.4 and 1.9 μm deepen systematically with increasing sphere diameter. However, these features coincide with strong atmospheric water-vapor absorption bands in the AM1.5 spectrum, where the terrestrial solar irradiance is strongly suppressed, and therefore contribute very little to the solar-weighted reflectance. This trend is consistent with experimental diffuse reflectance measurements of hydrated particulate materials, in which the strength of the 1.9 μm water-related absorption band increases with particle size.^20^ The remaining clear diameter-dependent differences occur beyond approximately 2.2 μm, where the solar irradiance is nonzero but small compared with the visible region. Consequently, the AM1.5-weighted reflectances span only 1.0 percentage point across the series and show no monotonic dependence on diameter. Thus, sphere diameter affects localized near-infrared spectral features but has little influence on the overall solar reflectance of these coatings. The apparent reflectance reduction of the coatings relative to the bare substrate may contain both physical losses—the increased optical path amplifying the small parasitic absorption of the nonideal substrate—and integrating-sphere collection effects arising from the comparison of a near-specular substrate and diffusely reflecting coatings against a diffuse BaSO_4_ reference; the relative reflectance of the bare substrate slightly exceeding 100% in the 400–500 nm region identifies this instrumental limitation. The measured reflectance is nevertheless sufficient to minimize solar absorption in all cases. Mid-infrared emissivity spectra [Figure 2(b)] show that the coatings achieve atmospheric-window emissivity (8–13 μm) between 0.91 and 0.95, in stark contrast to the bare substrate (0.02). This strong infrared emission arises from the intrinsic vibrational modes of SiO_2_ and the porous structure of the packed microsphere layer, which together produce broadband infrared emission even with a coating thickness of only 25 μm. The high emissivity within the atmospheric transparency window enables efficient radiative heat transfer to the sky.

To quantify the light transport regime, we performed coherent backscattering measurements on all coatings. Coherent backscattering is a manifestation of weak localization in disordered media,^21,22^ where constructive interference between time-reversed scattering paths produces an enhanced return signal precisely in the backscattering direction. The width of the coherent-backscattering cone provides a direct measure of the transport mean free path $l_{t}$ ,^23^ which characterizes the distance over which photons lose memory of their initial propagation direction and governs light diffusion in optically thick random media. Figure 3(a,b) shows a representative coherent-backscattering measurement for the 2 μm particle size coating; the two-dimensional maps of all samples are provided in Figure S6. The characteristic cone-shaped enhancement around zero detection angle is clearly visible, indicating strong multiple scattering. By fitting the angular profile to diffusion theory, we extract $l_{t,2\mu m}=\left(3.7\pm 0.2\right)$ μm. Repeating this analysis for all coatings yields the transport mean free paths shown in Figure 3(c) (black symbols). For the four cooling coatings, the measured values remain within a narrow band, $l_{t}\approx 3.7-5.7\;\mu m$ , so the coating thickness *L* ≈ 25 μm exceeds the transport length by factors $L/l_{t}\approx 4.4-6.8$ . These optical thicknesses establish multiple scattering, but they place the coatings in the transition between ballistic and diffusive transport rather than in a fully developed, semi-infinite diffusive regime, which requires substantially larger thicknesses.^24^ Moreover, because the layers have finite optical thickness and are deposited on a mirror that returns light into the scattering medium, internal reflection at the lower boundary modifies both diffusive transport and the coherent-backscattering line shape.^25^ The values reported here, extracted with the conventional semi-infinite expression, are therefore effective transport mean free paths of the complete coating–substrate geometry rather than intrinsic properties of the sphere packing; a finite-slab analysis including the reflecting boundary, together with a thickness-control experiment on an approximately 80 μm coating, is presented in the Supporting Information (Figure S7). For comparison, the red curve in Figure 3(c) shows the transport mean free path predicted by independent-scattering Mie theory, computed with no fitted scaling factor using a reference index *n* = 1.46 and an assumed filling fraction ϕ = 0.5—modeling assumptions rather than independently measured properties of each coating^26,27^ —and averaged over the measured 2% size distribution (Supporting Information, Section S3.1, Figure S5). The prediction is close to the measured value at the smallest diameter but grows steadily with *D*, exceeding the measurement by a factor of approximately 5–6 at *D* = 8 μm. This strongly diameter-dependent failure of the isolated-particle description is itself evidence of dependent scattering in these dense coatings, where the spheres are in permanent contact with several neighbors (nearest-neighbor center-to-center distance *r*_NN_ ≈ *D*): near-field coupling opens transport channels beyond any far-field description,^28^ structural correlations modify the transport mean free path,^29^ and measured transport in dense photonic glasses is not reproduced by single-particle theory.^30,31^ The polydispersity of the commercial microspheres, already accounted for in the model (Supporting Information), is a comparatively minor contribution. These measurements establish that all coatings are multiply scattering, with transport governed by collective, dependent scattering rather than by individual particle resonances, while the extracted transport lengths characterize the complete coating–substrate geometry.

**Figure 3:**
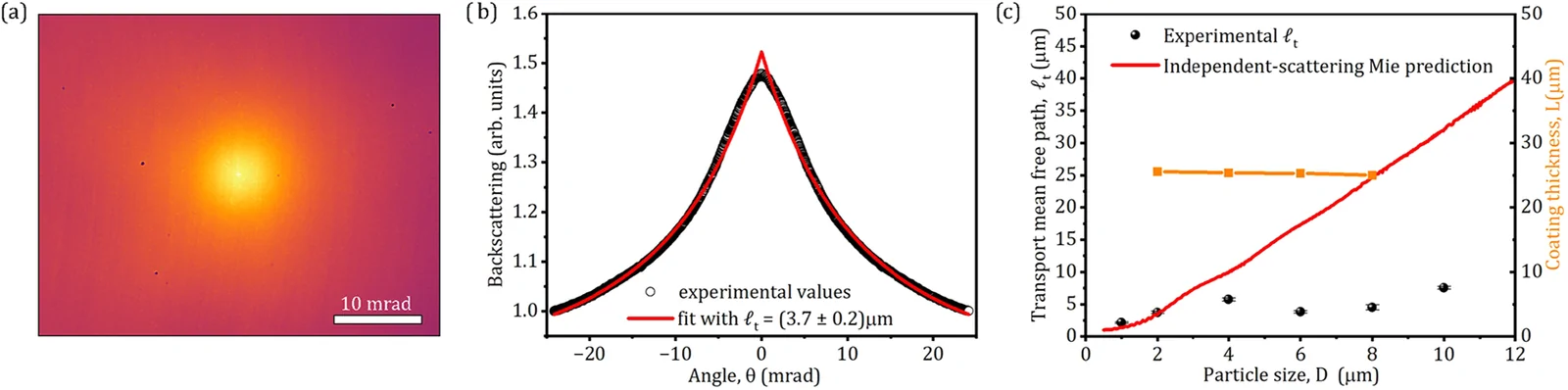
Light transport in disordered photonic coatings. (a) Two-dimensional CCD image of the backscattered intensity around the exact backscattering direction for the 2 μm coating, showing the interference-enhanced return intensity; the scale bar corresponds to 10 mrad. The maps of all samples are shown in Figure S6. (b) Coherent-backscattering profile (symbols) with fit (solid line) used to extract the transport mean free path $l_{t}$ ; the fit yields $l_{t}=\left(3.7\pm 0.2\right)$ μm for the 2 μm coating shown. (c) Measured transport mean free path $l_{t}$ (black symbols, with error bars) as a function of microsphere diameter *D*, compared with the independent-scattering Mie prediction computed with no fitted scaling factor (red curve). The coating thickness *L* ≈ 25 μm of the cooling coatings is shown as a separate series on the right axis (orange). The 1 and 10 μm data correspond to auxiliary photonic glass samples characterized only by coherent backscattering; no cooling coatings were fabricated from these particles. For the 2–8 μm cooling series, $L/l_{t}\approx 4.4-6.8$ , establishing multiple scattering in the ballistic-to-diffusive transition regime.

Despite the measurable variation in transport mean free path shown in Figure 3(c)—which varies nonmonotonically between approximately 3.7 and 5.7 μm across the series—the spectral optical properties remain remarkably consistent across all coatings. This is evident in Figure 2, where both solar reflectance over 250–2500 nm (93.8–94.8%) and atmospheric window emissivity (0.91–0.95) vary by less than approximately 3% across the full diameter range investigated. This weak diameter dependence of the complete coating–substrate architecture is a central finding of our work. The two spectral regions must, however, be distinguished. In the mid-infrared, the emissivity is governed primarily by intrinsic Si–O vibrational absorption in the approximately 25 μm silica layer, so the visible-range transport measurements are not invoked to explain its weak diameter dependence. In the solar range, the finite optical thickness permits a fraction of the light to reach the reflective substrate, which returns it through the scattering layer, making the total reflectance only weakly sensitive to the measured variation of $l_{t}$ . This finding is in contrast with previous theoretical results^18^ where the thermal emission and cooling response of periodic monolayers of identical particle sizes exhibit a pronounced diameter dependence. This effect was attributed to geometry-specific resonant effects happening only in the thin-layer limit. We next examine whether this spectral robustness translates into comparable cooling performance across the coatings.

To assess the macroscopic cooling behavior under operating conditions, coatings and ambient temperatures were continuously monitored, in addition to humidity, wind speed, solar radiation, and location. For the outdoor measurements reported in Figures 4 and 5, the temperature difference is defined as Δ*T* = *T*_s_ – *T*_a_, so negative values denote subambient cooling. As shown in Figure 4 (a), all photonic glass coatings remain at temperatures very close to ambient even under high solar irradiance (≈ 800 W m^–2^). Throughout the period of high irradiance, the coatings remain within approximately 2 °C of the ambient air [Figure 4(b)], and this difference decreases steadily during the afternoon, indicating an effective rejection of solar heating. In contrast, the highly reflective reference substrate—characterized by its high reflectance and low IR emissivity—remains consistently hotter than the ambient temperature during the same period. This behavior follows from the complete power balance. Despite its high solar reflectance, the bare substrate absorbs approximately (1 – 0.963) × 800 ≈ 30 W m^–2^ at the irradiance of the experiment, but its very low atmospheric-window emissivity (*ε* ≈ 0.02) provides almost no radiative heat-rejection channel; the absorbed load must therefore be dissipated predominantly through convection and conduction, requiring its temperature to rise above ambient. The silica coatings absorb more solar power, approximately 42–50 W m^–2^ (measured solar absorptance of 5.2–6.2%), but their high atmospheric-window emissivity provides a net radiative heat-rejection channel of comparable magnitude—the measured night-time cooling powers of 44–61 W m^–2^ at ambient temperature reported below—so their daytime energy budget balances near zero, and they remain close to ambient temperature. The coatings fall below ambient temperature during the second half of the afternoon, as the solar irradiance decreases. The photonic glass coatings reach a maximum temperature reduction of 4.8 °C relative to the reference substrate in the late afternoon. After sunset, the photonic glass coatings cool up to 3.7 °C below ambient, whereas the reference exhibits only minor cooling (approximately 0.5 °C). During the experiments, wind speeds ranged from 0 to 10 km h^–1^ and relative humidity from 30% to 60%. Although convective heat exchange with the air reduces the net radiative cooling effect, the coatings consistently outperform the reference.

**Figure 4:**
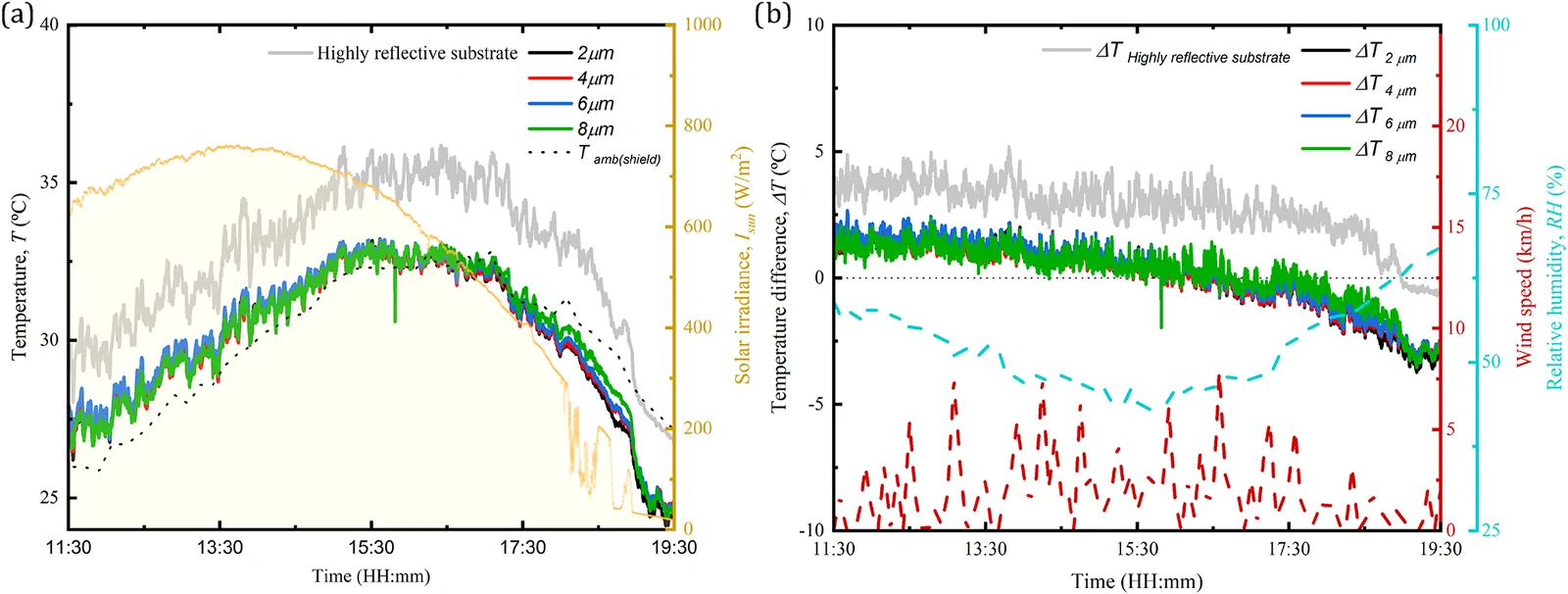
Thermal performance of photonic glass coatings. (a) Coating and reference temperatures *T* measured over an 8 h period from 11:30 to 19:30. Solar irradiance *I*_sun_ (yellow, right axis) shows peak values around 800 W m^–2^. All photonic glass coatings (colored lines) remain close to ambient temperature (Δ*T* ≈ 0), while the bare highly reflective substrate used as reference (gray) heats to more than 5 °C above ambient (Δ*T* > 5 °C). (b) Sample-to-ambient temperature difference, Δ*T* = *T*_s_ – *T*_a_, for the four coatings and the bare highly reflective substrate; negative values denote subambient cooling. Wind speed and relative humidity are shown on the right axes. The coatings successfully maintain near-ambient temperatures even under solar irradiation, confirming effective passive cooling.

**Figure 5:**
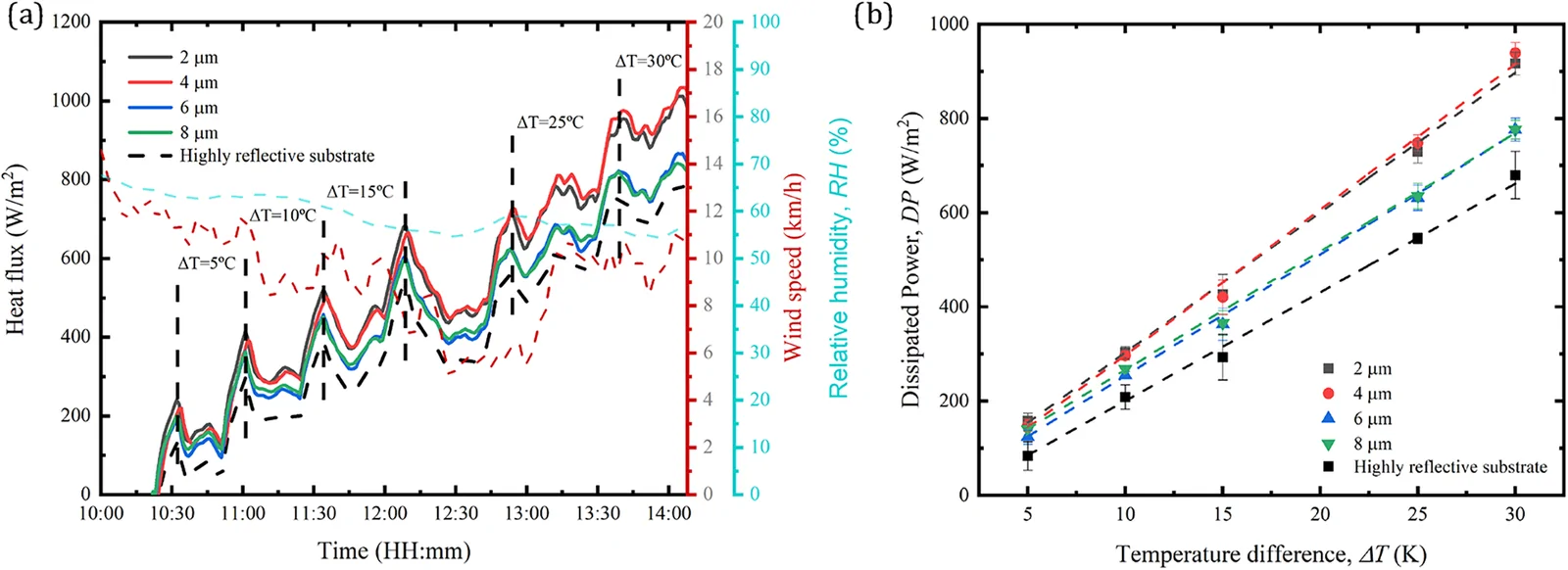
Dissipated power measurements. (a) Heat flux applied to the resistive heater at steps of Δ*T* = *T*_s_ – *T*_a_ = 5, 10, 15, 25, and 30 °C over a 4 h period for the four coatings (colored lines) and the bare highly reflective substrate (black dashed line). Stabilization was reached after each temperature step. Measurements were performed under monitored conditions of wind speed (red, right axis) and relative humidity (cyan, right axis). (b) Dissipated power delivered at each temperature step for the four coatings and the bare highly reflective substrate; the abscissa is expressed in K, numerically equal to the temperature difference in °C. Symbols correspond to the average heat flux value at each applied temperature step. Linear fit extrapolated to Δ*T* = 0 yields the intercept value.

To complement the temperature-based measurements, the cooling power of each coating was evaluated directly by monitoring the dissipated power (*DP*) or net cooling power capacity (*P*_net_) under outdoor conditions (see the Thermal Characterization: Dissipated Power section).^3,4^ The analysis is based on the energy balance described in eq 1

1 $$P_{\mathrm{net}}\;\left(T_{s},T_{a}\right)=P_{r}\;\left(T_{s}\right)-P_{a}\;\left(T_{a}\right)-P_{\mathrm{sun}}-P_{\mathrm{nonrad}}\;\left(T_{s},T_{a}\right)$$

where *P*_r_ is the thermal radiation emitted by the photonic glass coating, *P*_a_ is the absorbed atmospheric radiation, *P*_sun_ is the absorbed solar power, and *P*_nonrad_ accounts for nonradiative heat transfer processes such as convection and conduction, with *T*_s_ and *T*_a_ the sample and ambient temperatures, respectively. The electrical power supplied to a resistive heater was measured while sequentially imposing controlled temperature differences with respect to ambient (Δ*T* = 5–30 °C), as illustrated in Figure 5(a). At each step, the system reached thermal equilibrium, ensuring that the supplied electrical power balanced the total heat loss from the coating. The resulting linear dependence of *DP* on Δ*T*, shown in Figure 5(b), reflects the combined contribution of all the heat transfer mechanisms involved. Extrapolation to Δ*T* = 0 yields the baseline dissipated power under solar irradiation (daylight intercept values reported in Table 2, with an average uncertainty of ± 0.3 W m^–2^).

**Table 2:** Thermal Characterization Results for Photonic Glass Coatings^a^

coating	2 μm	4 μm	6 μm	8 μm
Daylight intercept (W m^–2^)	–0.1	0.5	0.2	–0.6
*P*_cool,night_ (W m^–2^)	60.8	46.2	44.1	54.8
Difference (W m^–2^)	60.9	45.7	43.9	55.4
Thermally estimated solar absorptance (%)	7.6	5.7	5.5	6.9
Measured absorptance, 250–2500 nm (%)	5.9	5.5	5.2	6.2
Measured absorptance, 250–900 nm (%)	6.1	5.5	5.0	5.7

a The daylight intercept values carry an average uncertainty of ± 0.3 W m^–2^; the outdoor energy balance has an estimated variability of ±20 W m^–2^.

It is important to note that, during the imposed temperature steps, both nonradiative heat transfer and radiative exchange with the atmosphere remain active and therefore contribute to the slope of the *DP*–Δ*T* linear fit. In theory, these temperature-dependent terms should vanish in the extrapolated limit Δ*T* = 0, where the conditions *h*_*c*_(*T*_s_ – *T*_a_) = 0 (convection and conduction) and σ­(*T*_s_^4^ – *T*_a_^4^) = 0 (radiative exchange with the opaque atmosphere) are fulfilled, where *h*_c_ is the effective convective/conductive heat transfer coefficient and σ is the Stefan–Boltzmann constant. However, the real atmosphere is not a blackbody slab at ambient air temperature. It is vertically stratified, spectrally selective, humidity-dependent, and more critically, its effective emitting temperature generally differs from near-surface ambient air temperature. As a consequence, the daylight intercept retains the nonvanishing radiative terms and the absorbed solar radiation, together with possible parasitic heat exchange through the test enclosure, mounts, and heater wiring, which is driven by temperature differences internal to the setup and therefore does not extrapolate to zero.

We complemented these measurements with night-time experiments under clear-sky conditions (approximately 20:20–22:00), with no solar irradiance and the heater set to maintain Δ*T* = 0. Under these conditions, the measured dissipated power (as shown in Figure S4 of the Supporting Information) reflects primarily radiative exchange with the sky (atmosphere and space), yielding a direct determination of the radiative cooling power (*P*_COOL,night_), as shown in Table 2. The *difference* between *P*_COOL,night_ and the daylight intercept approximates the net energy balance under solar exposure, assuming negligible differences in relative humidity. The daylight intercepts of the four coatings are comparable to their average uncertainty of ±0.3 W m^–2^, so their sign carries no physical significance: the small negative intercepts obtained for the 2 and 8 μm coatings are close to zero within the experimental variability and indicate an approximately balanced daytime energy budget, and no claim of daytime subambient cooling is made. However, deviations from the ideal energy balance arise from parasitic heat losses through the test box, convective effects, and local variations in outdoor conditions (e.g., wind, humidity, and cloud cover). These contributions result in a standard deviation of approximately ± 20 W m^–2^, corresponding to approximately 33–45% of the measured night-time cooling powers. The effective radiative sky temperature retrieved from these measurements, together with a consistency check of the energy balance model, is reported in the Supporting Information (Figure S4). Across the four coatings, these night-time cooling powers (44–61 W m^–2^) show no systematic dependence on microsphere diameter, consistent with the spectral robustness reported above. The 2 μm coating gives the highest night-time value (60.8 W m^–2^, compared with 44.1–54.8 W m^–2^ for the other coatings); because the samples were measured simultaneously, common atmospheric changes cannot explain this ordering, but the maximum difference of approximately 17 W m^–2^ is comparable to the ± 20 W m^–2^ variability, and the single-night measurement does not establish a reproducible particle-size dependence. At fixed packing fraction, the areal density of sphere–substrate contacts scales approximately as *D* ^–2^, which could favor interfacial heat transfer for the smallest spheres; however, the conductance of each contact depends on its area, load, roughness, adsorbed water, and surrounding air, none of which was measured independently, so this contact-density argument is presented only as a possible hypothesis and the reported cooling powers characterize the complete coating–substrate stack.

As an initial approximation, and assuming that nonradiative heat transfer mechanisms are negligible, the thermally estimated solar absorptance of the coatings can be estimated from the thermal measurements (reported as Thermally estimated solar absorptance in Table 2). The thermally derived values were subsequently normalized to standard conditions of 800 W m^–2^ and 25 °C in order to enable a meaningful comparison with the solar absorptance measured over the full 250–2500 nm solar range. The (measured) solar absorptance was calculated as *a*_total_ = 1 – *R*_material_, where *R*_material_ is the integrated reflectance obtained from eq 2. This normalization procedure is consistent with the linearized heat-balance treatment described in the Supporting Information, Section S2.3, where the measured dissipated power is interpreted through an effective loss coefficient that accounts for the combined convective and radiative contributions under the outdoor test conditions. By rescaling the thermal data to a common reference state, the thermally estimated and spectroscopically measured solar absorptances agree in magnitude, both lying in the 5–8% range. With the full-spectrum optical values, the thermal estimate exceeds the measured absorptance by only 0.2–1.7 percentage points, corresponding to approximately 2–14 W m^–2^ at the irradiance of the experiment—smaller than the estimated ± 20 W m^–2^ variability of the outdoor energy balance, so that neither the magnitude nor the sign of this difference is assigned physical significance. The residual discrepancy reflects the approximate nature of the thermal estimate, which remains subject to radiative exchange with the nonideal atmosphere and to parasitic heat exchange with the test enclosure. This broad consistency supports the reliability of the thermal measurements and the observed temperature-drop performance.

The experimental results presented here show that radiative cooling performance of photonic glass coatings remains largely stable across the investigated 2–8 μm range of particle diameters. This robustness is not a consequence of a fully developed diffusive regime: with $L/l_{t}\approx 4.4-6.8$ , the coatings are multiply scattering but operate in the transition between ballistic and diffusive transport.^24^ Instead, two distinct mechanisms underlie the weak diameter dependence of the complete coating–substrate architecture. In the mid-infrared, the emissivity in the ATW (8–13 μm) is governed primarily by the intrinsic Si–O vibrational absorption of the approximately 25 μm silica layer, and is therefore largely insensitive to the visible-range scattering properties of the individual spheres. In the solar range, the coatings scatter strongly, and the fraction of light that reaches the reflective substrate is returned through the scattering layer, so the total solar reflectance is only weakly sensitive to the measured, nonmonotonic variation of $l_{t}$ . As a result, changing the microsphere diameter alters the transport mean free path, but has little effect on the overall radiative response of the coating–substrate system. This interpretation is consistent with the observed robustness of the measured spectra, passive temperature reduction, and net cooling power, and aligns with previous studies of radiative cooling in optically thick disordered media.^7^ Importantly, this behavior relaxes the dependence of radiative cooling on precise particle-size control, within the investigated diameter range, coating thickness, and reflective-substrate-backed configuration.

In conclusion, we report passive radiative cooling using functionalizable photonic glass coatings, which maintain near-ambient temperatures under high solar irradiance and achieve subambient cooling in the late afternoon and after sunset, with net cooling powers lying within the same order of magnitude as those reported for state-of-the-art radiative coolers. By systematically varying the microsphere diameter and combining spectral, thermal, and power-based measurements across particle sizes spanning the visible and infrared scattering regimes, we find the cooling performance to be equal within experimental error. This robustness—observed for multiply scattering, reflective-substrate-backed coatings approximately 25 μm thick over the 2–8 μm diameter range—relaxes fabrication constraints and, crucially, decouples radiative cooling performance from precise control over particle size within this architecture. As a result, radiative cooling can be integrated with the full toolbox of colloidal chemistry, enabling functionalization, surface modification, and chemical tuning without compromising thermal performance, and opening pathways toward scalable, multifunctional cooling materials.

## Experimental Section

### Photonic Glass Coating Fabrication

Tested coatings were prepared by depositing polydisperse suspensions of commercial silica microspheres (Microparticles GmbH) with nominal diameters of 2, 4, 6, and 8 μm onto highly reflective substrates. The microspheres were dispersed in Milli-Q water and ultrasonicated before deposition; no surfactant, binder, or other additive was used. The commercial microspheres are solid, nonhollow amorphous SiO_2_ spheres. Coatings of 5 × 5 cm were fabricated using dispersions of 15 mL with concentrations of 0.4 wt % for coatings of 2 and 8 μm in diameter, and 0.5 wt % for 4 and 6 μm in diameter. These concentrations were identified as necessary to maintain a thickness of approximately 25 μm across all four particle sizes, with a standard deviation of ± 0.23 μm, as determined by mechanical profilometry. The common thickness of approximately 25 μm was fixed across the four samples to isolate the effect of microsphere diameter; substantially thicker particle coatings have also been explored in radiative cooling architectures,^32^ and a thickness-control experiment is presented in the Supporting Information (Figure S7). The substrates were placed in individual boxes of the same area and a controlled environment where the dispersion was allowed to settle naturally through a gravity-assisted process. Drying at 45 °C and 30% relative humidity yielded mechanically stable layers with excellent uniformity and a thickness of approximately 25 μm.

The substrate is a commercial highly reflective substrate supplied by Cooling Photonics S.L., consisting of an aluminum sheet coated with a multilayer optical stack comprising silver, SiO_2_, and TiO_2_: the silver layer provides broadband metallic reflectance, while the dielectric SiO_2_/TiO_2_ layers act as an interference coating that further enhances solar reflectance across the visible and near-infrared range. Its measured solar reflectance is 96.3% over 250–2500 nm, and its atmospheric-window emissivity is approximately 0.02. These spectral properties govern the radiative contribution of the substrate to the energy balance, while its thermal properties, the coating–substrate interface, and the mounting conditions may additionally affect transient and nonradiative heat transfer.

No glue, binder, or cohesive matrix was employed. Sphere–sphere cohesion in such binder-free silica assemblies is strongly humidity-dependent and arises primarily from capillary forces associated with adsorbed and interstitial water between neighboring silica surfaces, with weaker van der Waals interactions remaining after complete drying;^33–35^ similar short-range interactions likely contribute to the adhesion of the first particle layer to the substrate, although this interface was not characterized independently. The coatings remained intact during laboratory handling, transport, mounting, and the thermal outdoor measurements, but standardized adhesion tests were not performed, and resistance to direct rainfall was not investigated. Stability was assessed by repeated optical inspection; photographs of the four coatings taken on 6 days between 1 and 9 October 2025 during a week of continuous outdoor exposure in Madrid, an inland site, in uncovered Petri dishes left open to the ambient air, show localized wind damage on the 4 μm coating from 6 October onward, whereas the other three coatings show no visible cracking, delamination, or particle loss over the period documented (Supporting Information, Figure S8). The coatings used for the outdoor tests in Barcelona developed visible damage after a few additional nights of exposure following the reported thermal measurements. The night-time relative humidity at the coastal Barcelona site reaches 80–90% with dew formation, and we attribute this later degradation to repeated condensation and drying cycles acting on the capillary sphere–sphere cohesion, although this has not been tested independently; because the coatings are binder-free and unsealed, wind, rainfall, and humidity cycling could disturb the particle layer, and protective coatings are being investigated separately as a route toward practical outdoor deployment.

### Optical Spectroscopy

Reflectance over the 250–2500 nm solar range was obtained by combining UV–vis reflectance measurements (Cary 4000, Agilent Technologies, equipped with an Internal DRA 900 integrating sphere) with near-infrared reflectance measured by Fourier transform infrared spectroscopy (FTIR) spectroscopy equipped with an integrating sphere. A barium-sulfate-based diffuse reflectance standard was used as reference. Because the bare substrate is predominantly specular whereas the coatings reflect diffusely, the absolute comparison of both against the diffuse BaSO_4_ standard is affected by differences in angular collection; the relative reflectance of the bare substrate slightly exceeding 100% in the 400–500 nm region identifies this instrumental limitation. The spectrally weighted solar reflectance was calculated as

2 $$R\;\left(\%\right)=\frac{\int_{250}^{2500}I_{\mathrm{sun}}\;\left(\lambda \right)\;R_{\mathrm{material}}\;\left(\lambda \right)\;d\lambda }{\int_{250}^{2500}I_{\mathrm{sun}}\;\left(\lambda \right)\;d\lambda }$$

where *I*_sun_ is the AM1.5 global solar irradiance spectrum and *R*_material_ is the measured reflectance.

Mid-infrared emissivity was measured using FTIR spectroscopy (Vertex 80, Bruker) coupled with a gold-coated integrating sphere accessory (A562, Bruker), with a gold mirror as reference. Due to instrument availability, the 8 μm coating was measured using a Bruker Vertex 70 FTIR spectrometer equipped with a liquid-nitrogen-cooled MCT detector, whereas the remaining coatings were measured using the Bruker Vertex 80 system. The higher signal-to-noise ratio provided by the cooled MCT detector results in a smoother emissivity spectrum but does not affect the calculated emissivity values or the conclusions of this work. Since the coatings are optically opaque, emissivity equals absorptance *A* = 1 – *R* by Kirchhoff’s law. The atmospheric window emissivity was calculated as

3 $$\varepsilon =\frac{\int_{8}^{13}bb\;\left(\lambda ,T\right)\;A_{\mathrm{material}}\;\left(\lambda \right)\;d\lambda }{\int_{8}^{13}bb\;\left(\lambda ,T\right)\;d\lambda }$$

where bb­(λ, *T*) is the blackbody spectral radiance at temperature *T* and *A*_material_ = 1 – *R*_material_.

### Coherent Backscattering Measurements

Coherent-backscattering measurements were performed using a He–Ne laser source. The beam was spatially filtered to select the fundamental TEM_00_ mode, then directed through a beamsplitter onto the coating. A quarter-wave plate positioned between the beamsplitter and the coating created circular polarization for the incident light. Backscattered light passed through the same quarter-wave plate and beamsplitter before reaching a CCD camera. A linear polarizer placed before the camera filtered single-scattered photons (which have flipped helicity) while transmitting multiply scattered light (which randomizes polarization). The angular profile of the coherent backscattering cone was recorded and fitted to diffusion theory^23^ to extract the transport mean free path $l_{t}$ . The values so obtained are effective transport mean free paths of the complete coating–substrate geometry; a finite-slab analysis explicitly including the reflecting lower boundary^25^ is provided as a sensitivity analysis in the Supporting Information (Figure S7).

### Thermal Characterization: Temperature Drop

Temperature-drop measurements were performed under clear-sky outdoor conditions in Barcelona (Spain, October 2025). The sample-to-ambient temperature difference was calculated as Δ*T* = *T*_s_ – *T*_a_, with negative values denoting subambient cooling. Photonic glass coatings were mounted on a wooden platform covered with aluminized Mylar and oriented south, with sufficient spacing to avoid mutual shading (as shown in Figure S2 of the Supporting Information). The platform was fixed to a PVC tube anchored to a brick base, elevating the coatings 1.5 m above a flat concrete floor and at least 10 m away from any vertical structure to eliminate shading effects. Each coating was attached to its holder using double-sided adhesive tape and mounted on an expanded polystyrene support covered with a highly reflective, low-emissivity polymer film to minimize parasitic heat gains through the support structure. No side walls, additional thermal insulation, or convection-shield layers were used, ensuring an unobstructed view of the sky and allowing natural convective and radiative exchange with the surrounding environment. The configuration accommodated up to six coatings simultaneously, including the disordered silica sphere photonic glass coatings fabricated in this study and the bare highly reflective substrate used as the reference surface of the outdoor test.

Temperature measurements were performed using Class A, 4-wire PT100 probes (RSPro). Sensors monitoring coating temperature were bonded with conductive silver paint to the center of the backside of each coating. An additional sensor, housed in a Th-Friedrichs type 2033 radiation shield, measured ambient air temperature at the same height as the coatings and adjacent to a weather station (7002571 PC Weather Station, Bresser), which recorded relative humidity, wind speed, and other atmospheric parameters at 5 min intervals. Global horizontal irradiance was measured with a Class B pyranometer (CMP6, Kipp & Zonen) placed within 0.5 m of the setup. All temperatures and irradiance values were logged using a data acquisition system (DAQ970A, Keysight) with a sampling interval of 10 s.

### Thermal Characterization: Dissipated Power

Dissipated power was measured using an identical custom-built outdoor experimental system (as shown in Figure S3 of the Supporting Information). A resistive heating pad controlled by a PID loop implemented on a microcontroller maintained the coating surface at a fixed temperature set point above ambient, imposing a controlled temperature difference Δ*T* = *T*_s_ – *T*_a_ between the coating and the surrounding air. The electrical power supplied to the heater was continuously recorded and represents the energy required to offset heat losses from the coating. The night-time measurement used for the analysis in Section S2.4 of the Supporting Information was performed during a separate outdoor session on 11 November 2025.

## Supplementary Material



## Data Availability

The optical, thermal, and coherent-backscattering data underlying this study, together with the analysis scripts, are deposited at Zenodo: 10.5281/zenodo.22809643.

## Notes

SEM images of all four coatings; photographs of the outdoor temperature-drop and dissipated-power experimental setups; data-normalization procedure for the dissipated-power measurements; night-time heat flux measurements and retrieved effective sky temperature; independent-scattering Mie analysis of the transport mean free path, including single-particle resonance spectra; two-dimensional coherent-backscattering maps of all samples; and finite-slab coherent-backscattering analysis including the reflecting substrate boundary and a thickness-control experiment (PDF)
