# Supplementary material for: Robust Radiative Cooling in Functionalizable Silica Microsphere Paints
Source: ACS Photonics. 2026 Oct 19;13(21):6386–95. doi: 10.1021/acsphotonics.6c01355 (PMC13742292; doi:10.1021/acsphotonics.6c01355)
Supplement: Supplementary file 1 [file ph6c01355_si_001.pdf]

Supporting Information: Robust Radiative Cooling in Functionalizable Silica Microsphere Paints

Jorge Burgos,† José Rodrigo Magana,‡ Ares Lladós,¶ Javier Pascualena Ferré,¶ Sara Núñez-Sánchez,† Juliana Jaramillo Fernández,¶,‡ Cefe López,† and Pedro David García*,†

†*Instituto de Ciencia de Materiales de Madrid (ICMM), Consejo Superior de Investigaciones Científicas (CSIC), Calle Sor Juana Inés de la Cruz 3, 28049 Madrid, Spain*

‡*Cooling Photonics Sociedad Limitada, 08022 Barcelona, Spain*

¶*Departament de Màquines i Motors Tèrmics, Universitat Politècnica de Catalunya, 08028 Barcelona, Spain*

E-mail: pd.garcia@csic.es

S1 Coating Morphology

Figure S1 provides direct structural evidence for all four coatings. The microspheres are solid, non-hollow amorphous SiO_2 particles supplied by Microparticles GmbH. The images show broadly similar disordered morphologies across the series, but they are not used to infer an identical filling fraction for the four coatings. The common coating thickness of approximately 25 µm, with a standard deviation of ±0.23 µm across the series, was determined by mechanical profilometry rather than from the SEM images.

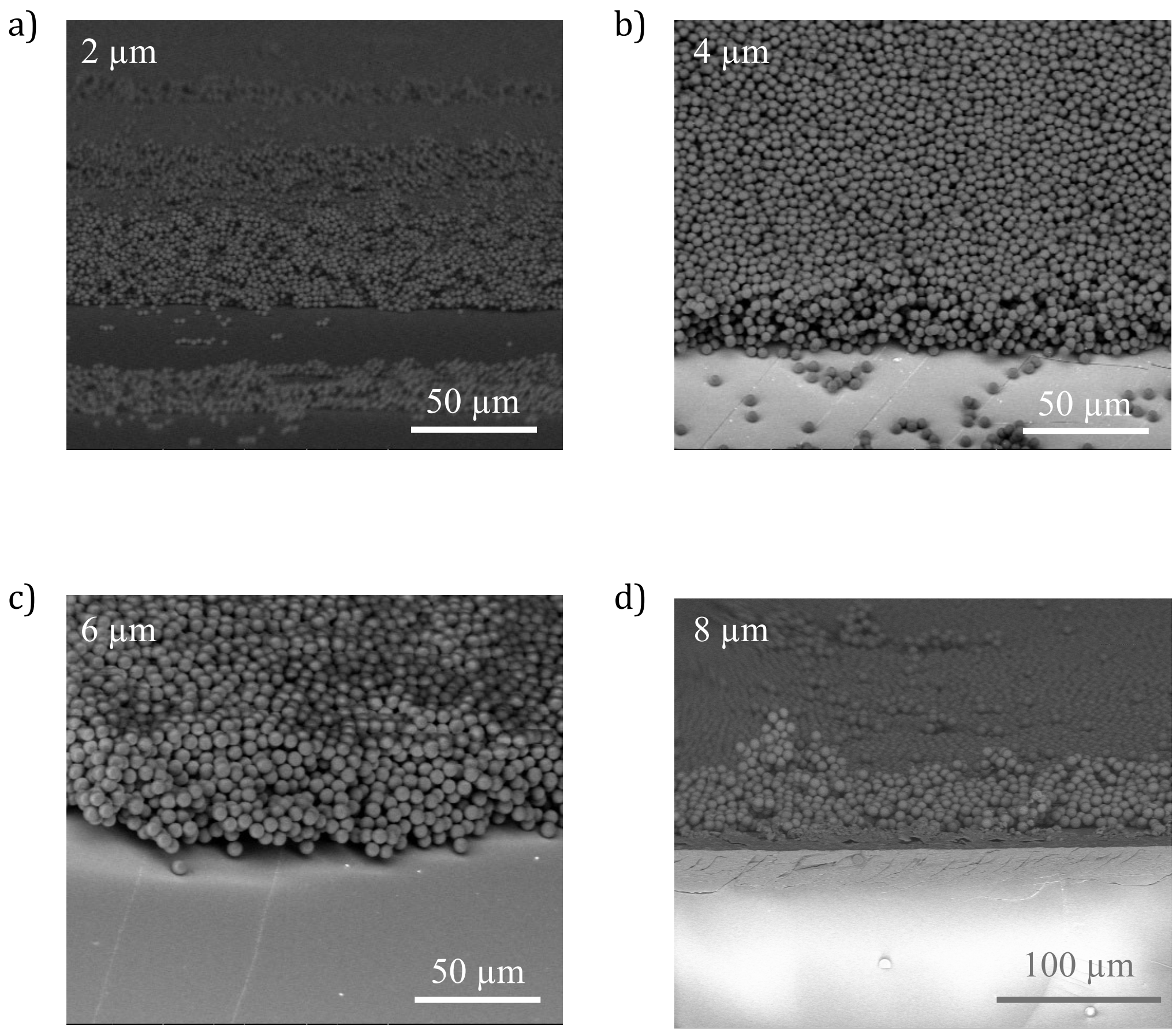

Figure S1: Scanning electron microscopy images of coatings prepared with nominal SiO_2 microsphere diameters of (a) 2, (b) 4, (c) 6, and (d) 8 µm. The top and edge views show the individual particles and their disordered, porous packing; the substrate is visible at the coating edges in panels (a)–(c). The scale bars are included in each panel.

S2 Thermal Characterization

S2.1 Temperature-drop measurements

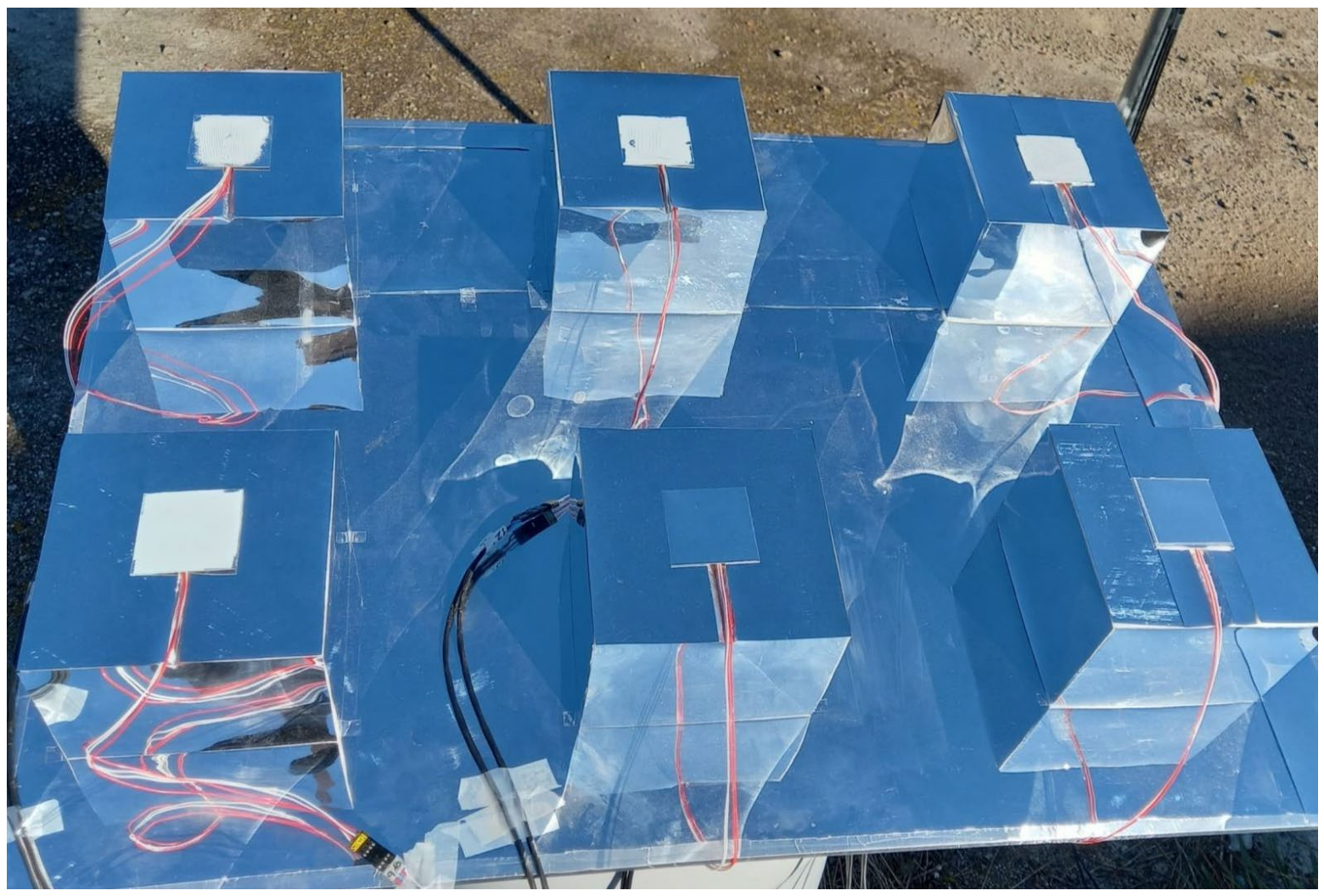

Figure S2: Outdoor platform used for the temperature-drop and cooling-performance measurements. The four photonic-glass coatings and the bare highly reflective substrate were mounted with sufficient spacing to avoid mutual shading.

Figure S2 shows the custom-built outdoor platform during the temperature-drop measurement. The four photonic-glass coatings are mounted side by side with the bare highly reflective substrate. No side walls, thermal insulation, or convection shields are used, so each sample exchanges heat with the sky and surrounding air under realistic, unobstructed conditions, while a low-emissivity support reduces thermal coupling to the platform. No power is supplied to the samples in this configuration. Throughout the thermal analysis, the temperature difference is defined as $\Delta T = T_\mathrm{s} - T_\mathrm{a}$, where T_s is the sample temperature and T_a is the ambient-air temperature; negative ΔT therefore denotes sub-ambient cooling. The back-side temperatures recorded under clear skies give the temperature differences reported in Figure 4 of the main text. The platform is reconfigured with a controlled resistive heater for the dissipated-power measurements described in Figure S3.

S2.2 Dissipated-power measurements

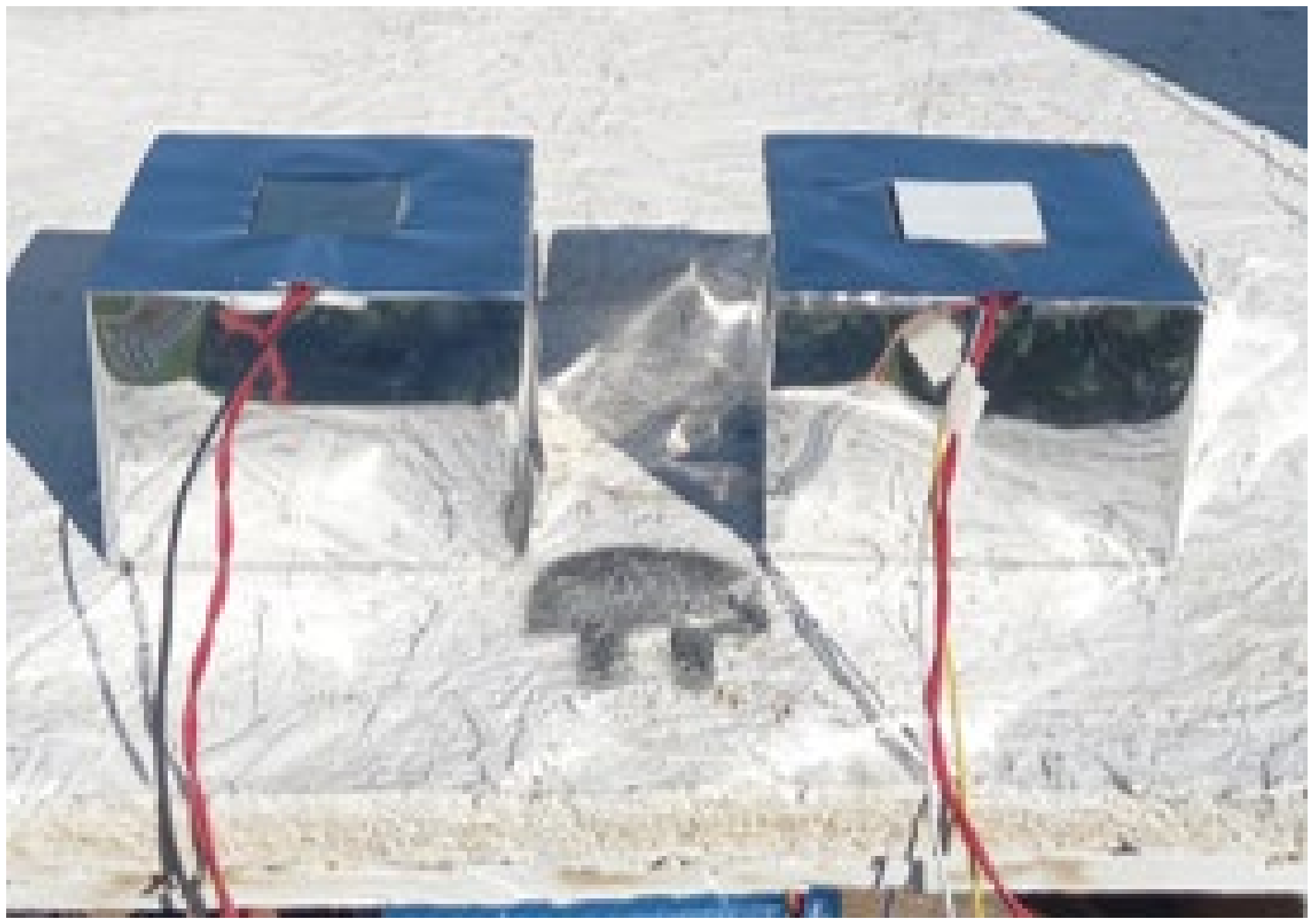

Figure S3: Dissipated-power experimental setup. A resistive heating pad attached to the back of each sample is controlled by a proportional–integral–derivative loop implemented on a microcontroller.

Figure S3 shows the dissipated-power configuration. A resistive heating pad bonded to the back of each sample is driven by a proportional–integral–derivative loop that holds the sample at a fixed temperature setpoint above ambient. For these dissipated-power measurements, $\Delta T = T_s - T_a$; the electrical power required to maintain a positive ΔT equals the net heat lost by the sample at that setpoint. Stepping ΔT and recording the supplied power after thermal equilibrium is reached yields the dissipated-power–ΔT relation shown in Figure 5 of the main text.

S2.3 Data normalization for dissipated-power measurements

Because the measurements depend on external conditions, including wind, ambient temperature, and solar irradiance, the data are normalized to fixed reference conditions. Each record contains the dissipated power P [$\mathrm{W\,m^{-2}}$], the plane-of-sample global solar irradiance G [$\mathrm{W\,m^{-2}}$], the ambient-air temperature T_a [K], and the sample surface temperature T_s [K],

together with the spectroscopic solar absorptance α and long-wave emissivity ε of the sample. The temperature difference is $\Delta T = T_\mathrm{s} - T_\mathrm{a}$. A steady, per-area daytime heat balance can be written as

$$P + \alpha G = h_\mathrm{c}\Delta T + q_\mathrm{LW} + q_\mathrm{par}, \tag{S1}$$

where h_c [$\mathrm{W\,m^{-2}\,K^{-1}}$] is an effective conduction/convection coefficient that includes wind and geometry, q_LW [$\mathrm{W\,m^{-2}}$] is the net long-wave exchange with the sky and surroundings, and q_par [$\mathrm{W\,m^{-2}}$] represents parasitic exchange through the test enclosure, mounts, and wiring. Positive terms on the right-hand side denote heat leaving the sample. This is the linearized form of the general energy balance introduced as Equation 1 in the main manuscript.

The net long-wave term is separated into its value at ambient sample temperature and its temperature-dependent part:

$$q_\mathrm{LW} \approx q_\mathrm{LW,0} + h_\mathrm{r}\Delta T. \tag{S2}$$

Here, $q_\mathrm{LW,0} = q_\mathrm{LW}(\Delta T = 0)$ is generally nonzero because the effective radiative temperature of the sky differs from the near-surface ambient-air temperature. For $\Delta T \lesssim 30$ K, the temperature-dependent part is linearized using

$$h_\mathrm{r} = 4\varepsilon\sigma T_\mathrm{m}^3, \qquad T_\mathrm{m} = \frac{T_\mathrm{s} + T_\mathrm{a}}{2}, \tag{S3}$$

where h_r is expressed in $\mathrm{W\,m^{-2}\,K^{-1}}$ and $\sigma = 5.670374419 \times 10^{-8}\ \mathrm{W\,m^{-2}\,K^{-4}}$. The mean-film linearization is accurate to within 1% for $\Delta T \lesssim 30$ K.

Normalization to a common ambient temperature and irradiance is performed at the same measured ΔT for each record. At the reference state,

$$T_\mathrm{s,ref} = T_\mathrm{a,ref} + \Delta T, \qquad T_\mathrm{m,ref} = \frac{T_\mathrm{s,ref} + T_\mathrm{a,ref}}{2}, \tag{S4}$$

and

$$h_{\mathrm{r,ref}} = 4\varepsilon\sigma T_{\mathrm{m,ref}}^3. \tag{S5}$$

The measured convective character of each record is preserved, while the absorbed solar load and the temperature-dependent radiative slope are referred to the common conditions. The normalized dissipated power is therefore written directly as

$$P_{\mathrm{ref}} = P + \alpha\,(G - G_{\mathrm{ref}}) + (h_{\mathrm{r,ref}} - h_{\mathrm{r}})\,\Delta T. \tag{S6}$$

Writing the correction in this form avoids assigning the nonzero atmospheric radiative offset to convection. Because the daytime atmospheric state and the parasitic exchange are not independently measured, their offsets are retained in the normalized data and contribute to the run-to-run variability rather than being forced to vanish.

For each sample, the normalized dissipated power is fitted as

$$P_{\mathrm{ref}} = P_0 + H\Delta T, \tag{S7}$$

where H is the measured effective slope and P_0 is the daylight intercept at $\Delta T = 0$. In terms of the balance above,

$$P_0 \approx q_{\mathrm{LW},0} + q_{\mathrm{par},0} - \alpha G_{\mathrm{ref}}. \tag{S8}$$

Thus, the intercept retains the non-vanishing radiative exchange with the real atmosphere and any parasitic exchange that survives at $\Delta T = 0$. A positive P_0 would indicate net sub-ambient cooling capacity under the reference daytime conditions. The measured intercepts are instead close to zero within the experimental variability, so no measurable daytime sub-ambient cooling is claimed. The intercept is an approximate energy-balance quantity and is not identified with the absorbed solar power alone.

As a power-balance consistency check, the bare substrate has a measured solar reflectance of 96.3% over 250–2500 nm, corresponding to an absorbed load of approximately

$(1-0.963)\times 800 \approx 30\ \mathrm{W\,m^{-2}}$ at $G = 800\ \mathrm{W\,m^{-2}}$. The coatings have measured full-spectrum solar absorptances of 5.2–6.2%, corresponding to approximately 42–50 $\mathrm{W\,m^{-2}}$. These values agree, within the estimated $\pm 20\ \mathrm{W\,m^{-2}}$ variability, with the approximate 44–61 $\mathrm{W\,m^{-2}}$ loads inferred from the difference between the night-time cooling power and the daylight intercept. The latter estimate combines measurements made under different atmospheric conditions and is used only as a consistency check.

At night, the short-wave term vanishes and $\Delta T \simeq 0$ suppresses surface-to-air convection and conduction. Neglecting residual parasitic exchange for the sky-temperature retrieval, the balance reduces to the radiative deficit relative to the effective sky:

$$P = \varepsilon\sigma\left(T_{\mathrm{a}}^4 - T_{\mathrm{sky}}^4\right). \tag{S9}$$

The effective radiative sky temperature is therefore

$$T_{\mathrm{sky}} = \left(T_{\mathrm{a}}^4 - \frac{P}{\varepsilon\sigma}\right)^{1/4}. \tag{S10}$$

The quantity $\Delta T_{\mathrm{sky}} = T_{\mathrm{a}} - T_{\mathrm{sky}}$ is the radiative contrast characterizing the nocturnal environment. Assuming this contrast is the descriptor carried between ambient conditions,

$$T_{\mathrm{sky,ref}} = T_{\mathrm{a,ref}} - \Delta T_{\mathrm{sky}},$$

and the normalized nocturnal requirement at $(T_{\mathrm{a,ref}}, G_{\mathrm{ref}} = 0)$ and $\Delta T_{\mathrm{ref}} = 0$ is

$$P_{\mathrm{ref,night}} = \varepsilon\sigma\left(T_{\mathrm{a,ref}}^4 - T_{\mathrm{sky,ref}}^4\right).$$

In practice, night-time rows are identified by low irradiance and near-zero ΔT. If measurement noise makes the inversion unstable, a clear-sky surrogate $T_{\mathrm{sky}} \approx T_{\mathrm{a}} - \Delta T_{\mathrm{sky,0}}$, with $\Delta T_{\mathrm{sky,0}}$ in the 10–20 K range, provides a stable fallback.

S2.4 Night-time heat flux and effective sky temperature

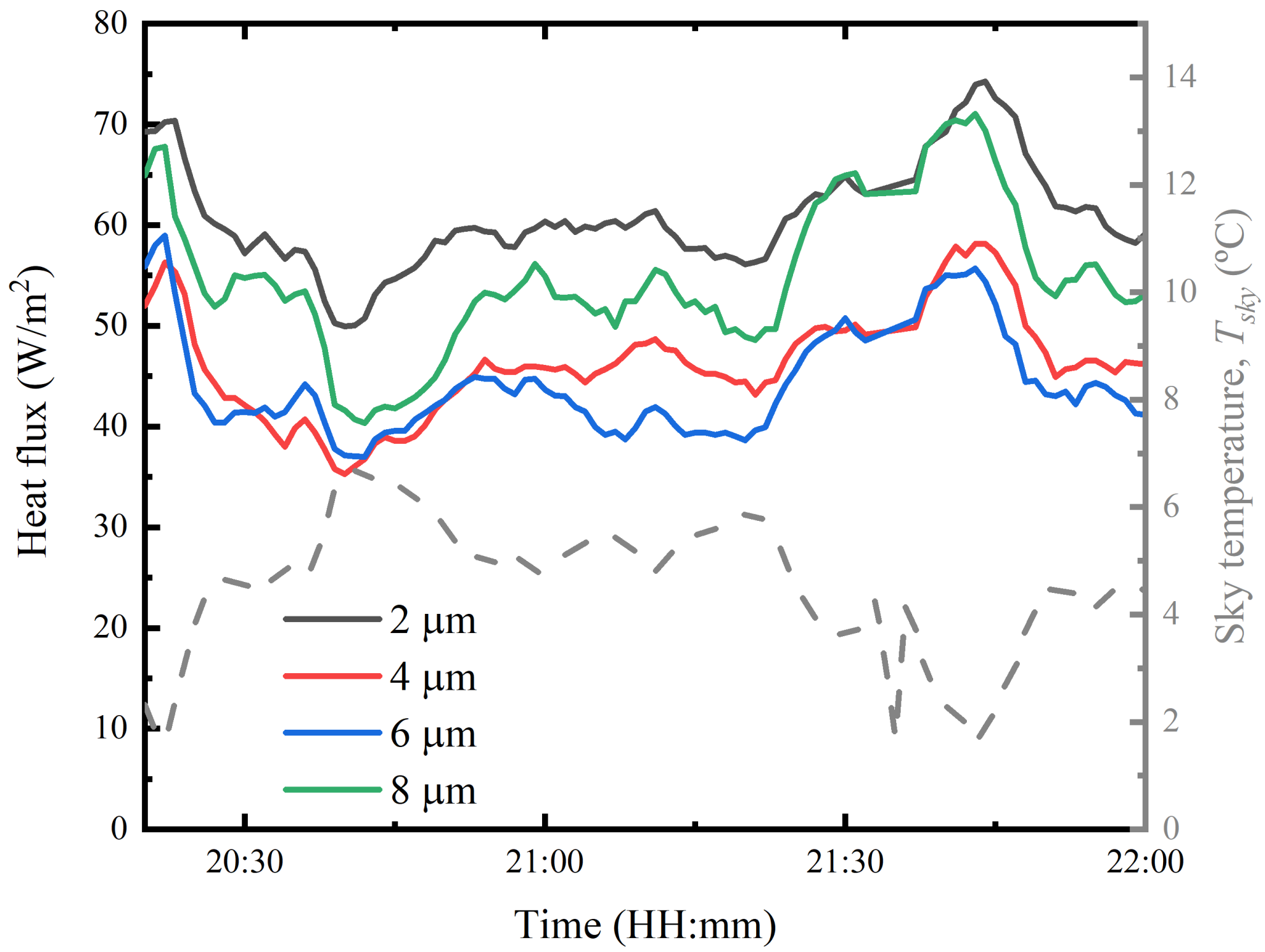

Figure S4: Night-time heat fluxes of the four coatings (solid curves, left axis) and mean effective radiative sky temperature T_{sky} (grey dashed curve, right axis) over the plotted interval, approximately 20:20–22:00. The PID loop maintains $\Delta T = T_{\text{s}} - T_{\text{a}} = 0$. The sky temperature is retrieved independently from each coating using Equation S10; the dashed curve shows their mean.

Figure S4 shows the night-time measurement under clear-sky conditions, with no solar irradiance and the PID loop holding $\Delta T = 0$. Surface-to-air convective and conductive terms are then suppressed to first order, so the applied heat flux reflects primarily the net radiative exchange between each coating and the sky. The resulting night-time cooling powers are 60.8, 46.2, 44.1, and 54.8 $\mathrm{W\,m^{-2}}$ for the 2, 4, 6, and 8 μm coatings, respectively. The 2 μm coating gives the highest value, but the maximum difference, 16.7 $\mathrm{W\,m^{-2}}$, is comparable to the estimated ±20 $\mathrm{W\,m^{-2}}$ variability of the outdoor energy balance. A single night-time measurement therefore does not establish a reproducible particle-size dependence.

Across the individual coating-based retrievals over the plotted interval in Figure S4, approximately 20:20–22:00, T_{sky} ranges from 274.8 to 281.9 K, with a mean over all coatings and times of 278.1 K. The four coatings provide independent retrievals because they were measured simultaneously; their standard deviation at each time is 1.7–2.8 K over the interval, with an average of 2.2 K. Substituting the cooling powers in Table II of the main text and the measured long-wave emissivities into Equation S9 with this sky temperature gives ambient temperatures of 287.4–290.6 K, mutually consistent within 3.3 K and representative of the measurement conditions. The resulting radiative contrasts are of order 10 K. Here, T_{sky} is an effective, spectrally integrated radiative temperature, not the physical air temperature at a particular altitude.

At fixed filling fraction, the areal density of sphere–substrate contacts scales approximately as D^{-2}, so the 2 µm coating has approximately sixteen times more contacts per unit area than the 8 µm coating. However, the conductance of each contact depends on its area, load, roughness, adsorbed water, and surrounding air. Neither the contact conductance nor heat transfer through the interstitial air was measured independently. Contact density is therefore only a possible hypothesis, and the reported cooling powers characterize the complete coating–substrate stack.

S3 Coherent Backscattering

S3.1 Independent-scattering calculation

The transport mean free paths ℓ_{t} reported in the manuscript were extracted from coherent-backscattering (CBS) measurements. For comparison, the independent-scattering prediction plotted in Figure 3(c) of the main text was recalculated without a fitted scaling factor. At the CBS wavelength $\lambda = 632.8$ nm, the calculation uses the transport cross-section of an isolated SiO_2 sphere in air, a reference refractive index $n = 1.46$, and an assumed particle

filling fraction $\phi = 0.5$:

$$\ell_{\mathrm{t}}^{\mathrm{Mie}} = \frac{1}{\rho\,\sigma_{\mathrm{tr}}}, \qquad \rho = \frac{6\phi}{\pi D^3}, \qquad \sigma_{\mathrm{tr}} = \langle \sigma_{\mathrm{s}}\,(1-g) \rangle\,. \tag{S11}$$

Here, ρ is the particle number density at filling fraction ϕ, σ_{s} is the single-particle scattering cross-section, $g = \langle \cos\theta \rangle$ is the anisotropy factor, and the angular brackets denote averaging of the transport cross-section $\sigma_{\mathrm{s}}(1-g)$ over the measured log-normal diameter distribution with 2% relative width. The refractive index and filling fraction are modelling assumptions rather than independently measured properties of every particle batch or coating. The corresponding scattering efficiency Q_{sca} and transport efficiency $Q_{\mathrm{tr}} = Q_{\mathrm{sca}}(1-g)$ are shown in Figure S5. Polydispersity suppresses the sharpest monodisperse Mie resonances while retaining their broad spectral envelope.

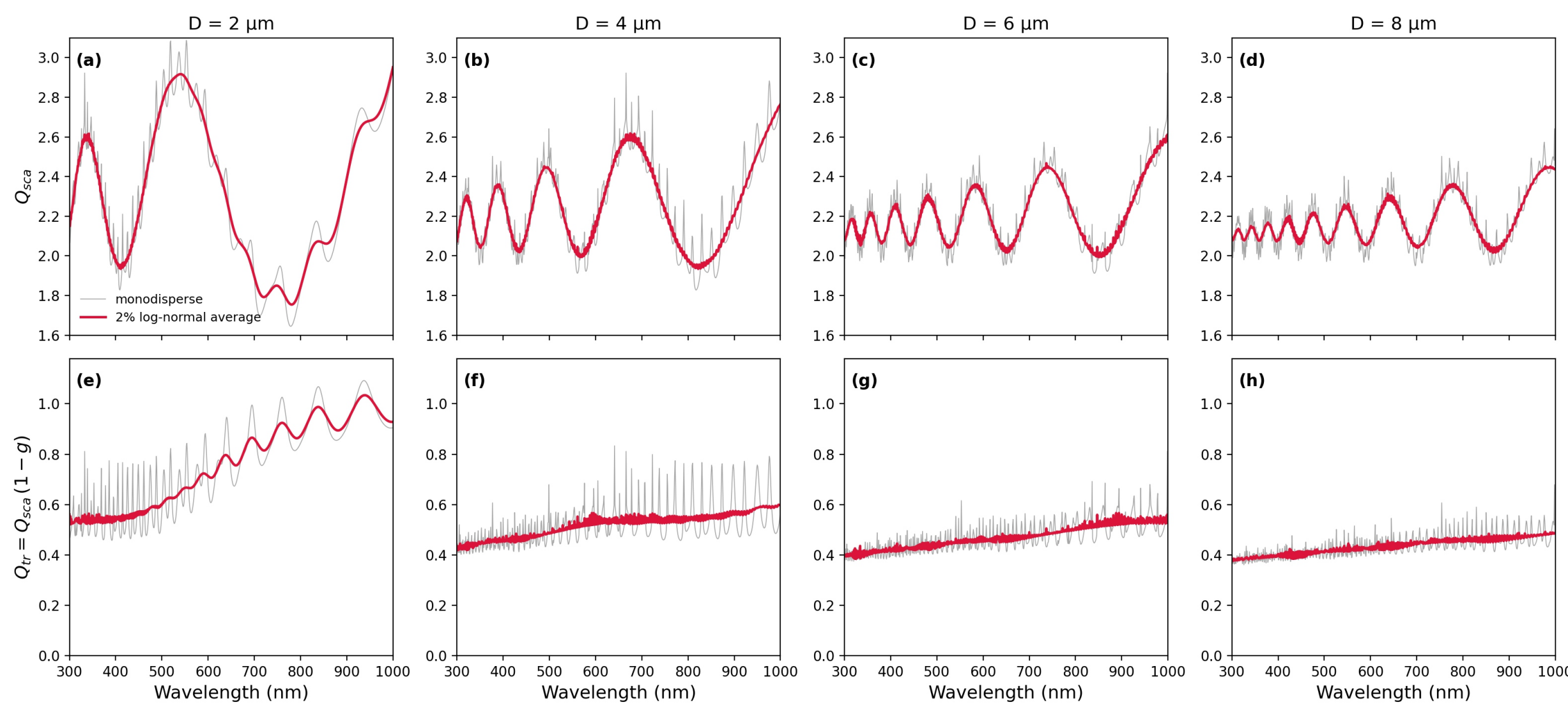

Figure S5: Single-particle Mie calculations for SiO_2 spheres with nominal diameters $D = 2$, 4, 6, and 8 µm in air. Panels (a)–(d) show the scattering efficiency Q_{sca}; panels (e)–(h) show the transport efficiency $Q_{\mathrm{tr}} = Q_{\mathrm{sca}}(1-g)$. Grey curves are monodisperse calculations and red curves are averages over the measured 2% log-normal diameter distribution. The calculations use the same $n = 1.46$ reference index as Equation S11.

The independent-scattering prediction is close to the measured value at the smallest diameter but increases strongly with D and exceeds the measurement by a factor of approx-

imately 5–6 at $D = 8$ µm. By contrast, the measured values for the four cooling coatings remain within $\ell_t \approx 3.7$–5.7 µm, corresponding to $L/\ell_t \approx 4.4$–6.8 for $L \approx 25$ µm. Thus, the isolated-particle calculation does not quantitatively describe transport in these dense coatings.

In a far-field dependent-scattering description, positional correlations enter through the static structure factor $S(q)$:[S2]

$$\ell_t^{-1} = \frac{\pi\rho}{k^6}\int_0^{2k} F(q)S(q)q^3\,\mathrm{d}q, \tag{S12}$$

where k is the wavenumber in the effective medium, $q = 2k\sin(\theta/2)$ is the scattering-vector magnitude, $F(q) = k^2\,(\mathrm{d}\sigma/\mathrm{d}\Omega)(q)$ is the dimensionless single-particle Mie form factor, with $\mathrm{d}\sigma/\mathrm{d}\Omega$ the differential scattering cross-section, and $S(q)$ describes the positional correlations of the particles. This construction has been tested in concentrated sphere suspensions,[S3] but the true three-dimensional $S(q)$ of the present dry, polydisperse coatings is not available. We therefore do not evaluate the $F(q)S(q)$ integral using an assumed analytical form for $S(q)$ and do not apply a global multiplicative correction. In addition, near-field coupling in dense media introduces transport channels not contained in a far-field $F(q)S(q)$ treatment.[S4] The measured disagreement with the independent-scattering result is consistent with previous studies of resonant transport in dense photonic glasses[S5,S6] and with the broadened scattering produced by closely spaced, hierarchically organized SiO_2 particles.[S7]

Packing density and filling fraction describe the same structural quantity ϕ, whereas the interparticle porosity is $1 - \phi$; both are distinct from internal microporosity within the silica spheres. Internal porosity can reduce the effective particle refractive index below that of dense fused silica.[S8] All independent-scattering calculations in this work use a single value, $\phi = 0.5$. The filling fraction of the present coatings was not measured; $\phi = 0.5$ was chosen because the coatings form by sedimentation from dilute suspensions (0.4–0.5 wt%), for which experimental studies of sphere sediments report $\phi \approx 0.55$ for hard spheres,[S13] $\phi \approx 0.49$ for

charge-stabilized silica,[S14] and a gravimetric value of 0.53 for a photonic-glass film;[S15] these values lie below the random-close-packing limit of approximately 0.64.[S9] At fixed D and n, Equation S11 gives $\ell_t^{Mie} \propto \phi^{-1}$, so over the resulting range $\phi = 0.49$–0.55 the calculated ℓ_t^{Mie} varies by at most 12% relative to its value at $\phi = 0.5$, far less than the factor-of-5–6 discrepancy with the measured transport mean free path at $D = 8$ µm (Figure 3c of the main text). Reducing n below 1.46 would weaken the isolated-particle scattering and increase ℓ_t^{Mie}, widening rather than removing that discrepancy.

The SEM images show frequent particle contacts, so the nearest-neighbour centre-to-centre distance can be approximated as $r_{NN} \approx D$, with an approximately zero local surface gap at contacts. The complete gap distribution and the filling fraction of each coating were not measured. Their effects enter through $S(q)$ and other dependent-scattering contributions and are incorporated empirically in the measured CBS transport lengths. The normalized two-dimensional CBS maps of all measured samples, including the auxiliary 1 and 10 µm photonic glasses, are shown in Figure S6.

S3.2 Finite-slab model and reflecting lower boundary

The conventional CBS analysis used for the complete diameter series employs the standard semi-infinite diffusive line shape.[S1] Because the present layers have finite optical thickness and are deposited on a mirror, we additionally fitted the 25 and 80 µm coatings made from 8 µm spheres with a finite-slab model that explicitly includes the reflecting lower boundary (Figure S7). This is a sensitivity analysis of the boundary condition; the conventional values are retained in the main text for consistency across the complete diameter series.

The reflecting-boundary fits multiply the conventional semi-infinite line shape[S1] by an angle-dependent correction factor obtained from the depth-integrated diffuse propagator of a slab of thickness L, with $z = 0$ at the air–coating interface and $z = L$ at the coating–substrate interface, using the standard exponential weights for the first and last scattering events. The air side is an open boundary with extrapolation length $z_e = 2\ell_t/3$, and the reflecting

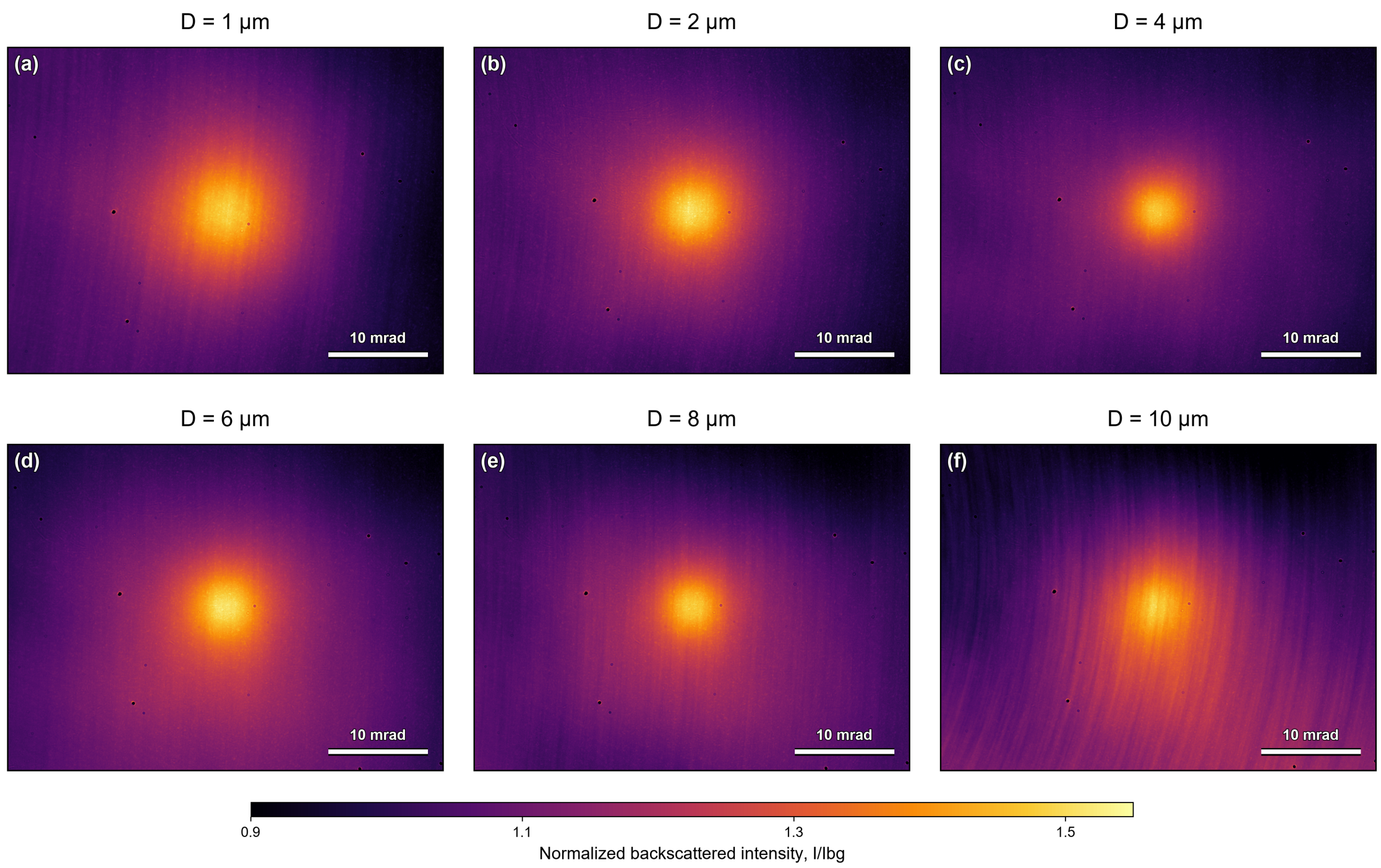

Figure S6: Normalized two-dimensional coherent-backscattering maps for coatings prepared with nominal microsphere diameters of (a) 1, (b) 2, (c) 4, (d) 6, (e) 8, and (f) 10 μm. All panels use the same normalized-intensity colour scale and contain a 10 mrad scale bar. The 1 and 10 μm samples are auxiliary CBS measurements; the radiative-cooling series comprises the 2, 4, 6, and 8 μm coatings.

substrate enters through the simplified mixed, or Robin, boundary condition of Lagendijk, Vreeker, and De Vries (LVdV),[S11] with extrapolation ratio $\epsilon = R/(1-R)$ evaluated at the measured substrate reflectance $R = 0.976$ at 632.8 nm ($\epsilon = 40.7$), so that the lower boundary is close to flux-free. The correction factor is the ratio of the reflecting-slab propagator to the open-slab ($R = 0$) propagator and therefore reduces exactly to the conventional line shape for a non-reflecting substrate. In the fits, L is fixed to the measured coating thickness, R is fixed to 0.976, and ℓ_t is the only transport parameter varied in the cone line shape; the remaining free parameters set only the overall normalization and the incoherent background. The mirror therefore modifies the cone through a specified boundary condition, with no adjustable rescaling parameter.

For the 25 µm coating, the conventional fit gives $\ell_t = (4.53 \pm 0.40)$ µm and $L/\ell_t = 5.5$, whereas the reflecting-boundary fit gives $\ell_t = (2.63 \pm 0.35)$ µm and $L/\ell_t = 9.5$. For the 80 µm coating, the corresponding values are (7.20 ± 0.40) µm and 11.1 without the mirror boundary, and (5.35 ± 0.40) µm and 14.9 with it. The correction is therefore approximately 26–42%, and the difference between the two coating thicknesses persists under both boundary treatments. The observation of well-defined cones shows multiple scattering, but the conventional extraction does not yield a thickness-independent bulk transport length. The reported values are therefore effective transport mean free paths of the corresponding coating–substrate geometries rather than intrinsic properties of the sphere packing. This interpretation is consistent with the ballistic-to-diffusive transition expected for finite scattering slabs.[S10]

The 8 µm spheres were selected for the thickness control because only approximately three particle diameters span the original 25 µm layer, whereas the 80 µm layer accommodates approximately ten diameters and can develop a more bulk-like interior. Consequently, a genuine difference in local packing may contribute in addition to the boundary-model sensitivity, and the present measurements cannot separate those effects. The thicker layer is included only as a transport control; the radiative-cooling results are obtained directly from the original 25 µm coatings. Substantially thicker particle coatings have also been explored

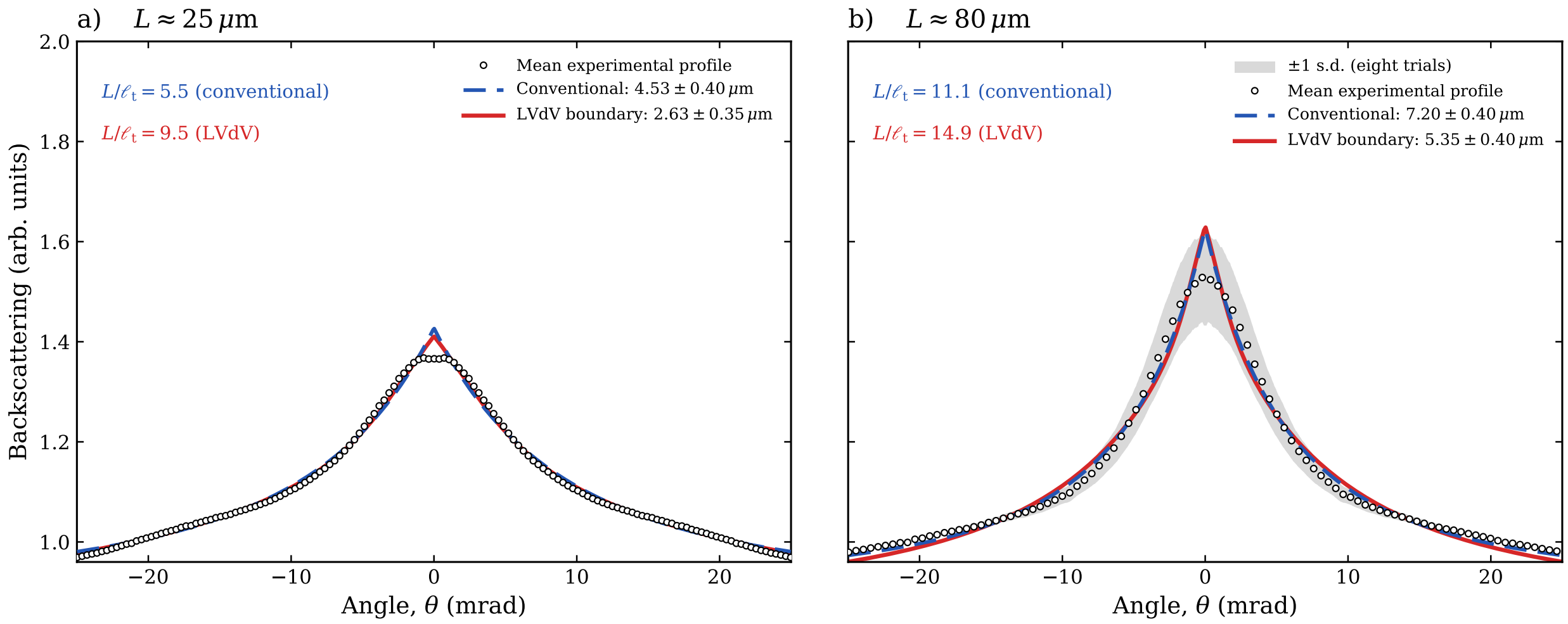

Figure S7: Thickness-control CBS measurements for coatings made with 8 µm microspheres: (a) $L \approx 25$ µm and (b) $L \approx 80$ µm. Black circles show the mean experimental profiles; the grey band in panel (b) shows ± 1 standard deviation over eight trials. Blue dashed curves are the conventional semi-infinite fits, which give $\ell_t = (4.53 \pm 0.40)$ and (7.20 ± 0.40) µm, corresponding to $L/\ell_t = 5.5$ and 11.1. Red curves are finite-slab fits using the reflecting LVdV lower boundary with the measured $R = 0.976$; they give $\ell_t = (2.63 \pm 0.35)$ and (5.35 ± 0.40) µm, corresponding to $L/\ell_t = 9.5$ and 14.9.

in radiative-cooling architectures.[S12]

S4 Mechanical Stability

Stability of the coatings was assessed by repeated optical inspection. Figure S8 shows photographs of the four coatings taken on six days between 1 and 9 October 2025 during a week of continuous outdoor exposure in Madrid, an inland site, in uncovered Petri dishes. The coatings were open to the ambient air throughout; the adhesive tape visible in the photographs secures the dishes to their support and does not cover the coatings. Localized wind damage is visible on the 4 µm coating from 6 October onward, while the other three coatings show no visible cracking, delamination or particle loss over the period documented. As discussed in the Methods of the main text, the coatings used for the outdoor tests in Barcelona developed visible damage after a few additional nights of exposure following the reported measurements, which we attribute to condensation and drying cycles under the high

night-time humidity of the coastal site; standardized adhesion tests were not performed.

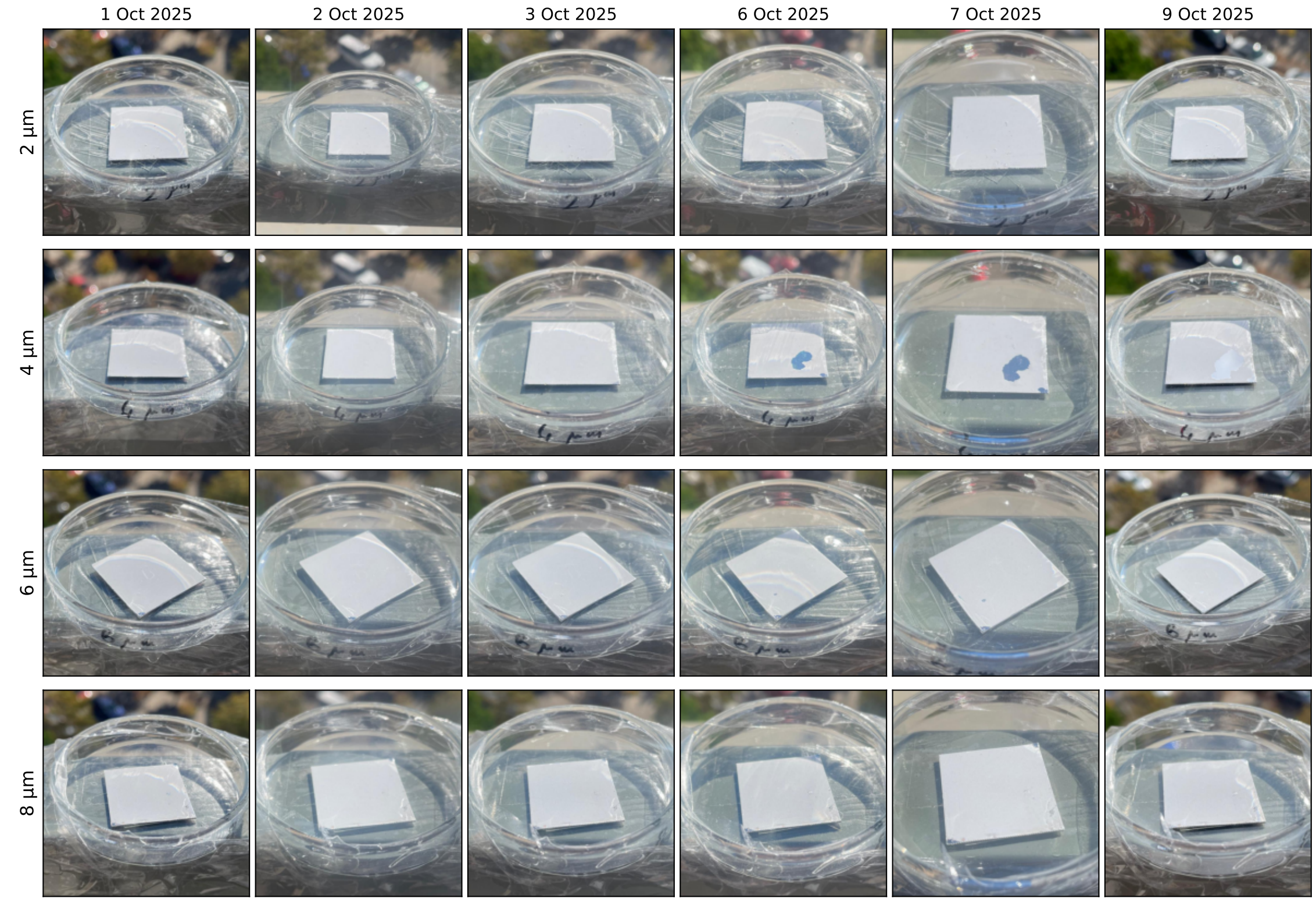

Figure S8: Optical photographs of the photonic glass coatings with nominal microsphere diameters of 2, 4, 6 and 8 µm (rows) on six days between 1 and 9 October 2025 (columns) during continuous outdoor exposure in Madrid in uncovered Petri dishes, open to the ambient air throughout; the adhesive tape visible in the photographs secures the dishes and does not cover the coatings. Localized damage caused by wind is visible on the 4 µm coating from 6 October onward, whereas no visible damage is observed in the other three coatings over the period documented.

References

(S1) E. Akkermans, P. E. Wolf, and R. Maynard, Coherent backscattering of light by disordered media: Analysis of the peak line shape. *Phys. Rev. Lett.* **1986**, *56*, 1471–1474. DOI: 10.1103/PhysRevLett.56.1471.

(S2) K. Vynck, R. Pierrat, R. Carminati, L. S. Froufe-Pérez, F. Scheffold, R. Sapienza,

S. Vignolini, and J. J. Sáenz, Light in correlated disordered media. *Rev. Mod. Phys.* **2023**, *95*, 045003. DOI: 10.1103/RevModPhys.95.045003.

(S3) S. Fraden and G. Maret, Multiple light scattering from concentrated, interacting suspensions. *Phys. Rev. Lett.* **1990**, *65*, 512–515. DOI: 10.1103/PhysRevLett.65.512.

(S4) R. Rezvani Naraghi, S. Sukhov, J. J. Sáenz, and A. Dogariu, Near-field effects in mesoscopic light transport. *Phys. Rev. Lett.* **2015**, *115*, 203903. DOI: 10.1103/PhysRevLett.115.203903.

(S5) P. D. García, R. Sapienza, J. Bertolotti, M. D. Martín, Á. Blanco, A. Altube, L. Viña, D. S. Wiersma, and C. López, Resonant light transport through Mie modes in photonic glasses. *Phys. Rev. A* **2008**, *78*, 023823. DOI: 10.1103/PhysRevA.78.023823.

(S6) D. Montesdeoca, F. Bayat, A. Espinha, Á. Blanco, C. Pecharromán, and C. López, Monodisperse silica spheres ensembles with tailored optical resonances in the visible. *Part. Part. Syst. Charact.* **2016**, *33*, 871–877. DOI: 10.1002/ppsc.201600194.

(S7) Y. Ding, Q. Zhou, X. Li, Y. Xiong, and S. Guo, PC light-scattering material containing "pomegranate-like" SAN–SiO_2 microspheres with excellent effective scattering range based on large-screen display. *Compos. Sci. Technol.* **2021**, *201*, 108532. DOI: 10.1016/j.compscitech.2020.108532.

(S8) F. García-Santamaría, H. Míguez, M. Ibisate, F. Meseguer, and C. López, Refractive index properties of calcined silica submicrometer spheres. *Langmuir* **2002**, *18*, 1942–1944. DOI: 10.1021/la010813g.

(S9) H. M. Jaeger, S. R. Nagel, and R. P. Behringer, Granular solids, liquids, and gases. *Rev. Mod. Phys.* **1996**, *68*, 1259–1273. DOI: 10.1103/RevModPhys.68.1259.

(S10) R. Elaloufi, R. Carminati, and J.-J. Greffet, Diffusive-to-ballistic transition in dynamic

light transmission through thin scattering slabs: A radiative transfer approach. *J. Opt. Soc. Am. A* **2004**, *21*, 1430–1437. DOI: 10.1364/JOSAA.21.001430.

(S11) A. Lagendijk, R. Vreeker, and P. de Vries, Influence of internal reflection on diffusive transport in strongly scattering media. *Phys. Lett. A* **1989**, *136*, 81–88. DOI: 10.1016/0375-9601(89)90683-X.

(S12) W. Huang, Y. Chen, Y. Luo, J. Mandal, W. Li, M. Chen, C.-C. Tsai, Z. Shan, N. Yu, and Y. Yang, Scalable aqueous processing-based passive daytime radiative cooling coatings. *Adv. Funct. Mater.* **2021**, *31*, 2010334. DOI: 10.1002/adfm.202010334.

(S13) S. R. Liber, S. Borohovich, A. V. Butenko, A. B. Schofield, and E. Sloutskin, Dense colloidal fluids form denser amorphous sediments. *Proc. Natl. Acad. Sci. U.S.A.* **2013**, *110*, 5769–5773. DOI: 10.1073/pnas.1214945110.

(S14) P. M. Nanikashvili, A. V. Butenko, S. R. Liber, D. Zitoun, and E. Sloutskin, Denser fluids of charge-stabilized colloids form denser sediments. *Soft Matter* **2014**, *10*, 4913–4921. DOI: 10.1039/c4sm00128a.

(S15) V. Hwang, A. B. Stephenson, S. Magkiriadou, J.-G. Park, and V. N. Manoharan, Effects of multiple scattering on angle-independent structural color in disordered colloidal materials. *Phys. Rev. E* **2020**, *101*, 012614. DOI: 10.1103/PhysRevE.101.012614.
